# Altered Steroidome in Women with Multiple Sclerosis

**DOI:** 10.3390/ijms252212033

**Published:** 2024-11-08

**Authors:** Radmila Kancheva, Martin Hill, Marta Velíková, Ludmila Kancheva, Josef Včelák, Radek Ampapa, Michal Židó, Ivana Štětkářová, Jana Libertínová, Michala Vosátková, Eva Kubala Havrdová

**Affiliations:** 1 Institute of Endocrinology, 11000 Prague, Czech Republic; mvelikova@endo.cz (M.V.); lkancheva@endo.cz (L.K.); jvcelak@endo.cz (J.V.); mvosatkova@endo.cz (M.V.); 2 MS Center, Jihlava Hospital, 58633 Jihlava, Czech Republic; ampapar@gmail.com; 3 Department of Neurology 3FM CU and UHKV, Third Faculty of Medicine, Charles University, 10000 Prague, Czech Republic; michal.zido@fnkv.cz (M.Ž.); ivana.stetkarova@fnkv.cz (I.Š.); 4 MS Center, Second Faculty of Medicine, Charles University, 15006 Prague, Czech Republic; jana.libertinova@fnmotol.cz; 5 Department of Neurology, First Faculty of Medicine, Charles University, 12008 Prague, Czech Republic; ehavr@lf1.cuni.cz

**Keywords:** steroidomics, multiple sclerosis, GC-MS/MS, multivariate statistics, differential diagnostics

## Abstract

Multiple sclerosis (MS) is a chronic inflammatory disease of the central nervous system (CNS) mainly afflicting young women. Various steroids can influence the onset and development of the disease or, on the contrary, mitigate its course; however, a systematic review of steroidomic changes in MS patients is lacking. Based on the gas chromatography tandem mass spectrometry (GC-MS/MS) platform and, in the case of estradiol, also using immunoassay, this study performed a comprehensive steroidomic analysis in 25 female MS patients aged 39(32, 49) years compared to 15 female age-matched controls aged 38(31, 46) years. A significant trend towards higher ratios of conjugated steroids to their unconjugated counterparts was found in patients, which is of particular interest in terms of the balance between excitatory and inhibitory steroid modulators of ionotropic receptors. Patients showed altered metabolic pathway to cortisol with decreased conversion of pregnenolone to 17-hydroxypregnenolone and 17-hydroxypregnenolone to 17-hydroxyprogesterone and increased conversion of 17-hydroxypregnenolone to dehydroepiandrosterone (DHEA), resulting in lower levels of 17-hydroxyprogesterone, as well as indications of impaired conversion of 11-deoxy-steroids to 11β-hydroxy-steroids but reduced conversion of cortisol to cortisone. Due to over-activation of hypothalamic-pituitary-adrenal axis (HPAA), however, cortisol and cortisone levels were higher in patients with indications of depleted cortisol synthesizing enzymes. Patients showed lower conversion of DHEA to androstenedione, androstenedione to testosterone, androstenedione to estradiol in the major pathway, and testosterone to estradiol in the minor pathway for estradiol synthesis at increased conversion of androstenedione to testosterone. They also showed lower conversion of immunoprotective Δ^5^ androstanes to their more potent 7α/β-hydroxy metabolites and had lower circulating allopregnanolone and higher ratio 3β-hydroxy-steroids to their neuroprotective 3α-hydroxy-counterparts.

## 1. Introduction

Multiple sclerosis (MS) is a chronic inflammatory disease of the central nervous system (CNS) that afflicts young people and results in demyelination and neurodegeneration. Multiple sclerosis is more common in women, with a current female-to-male ratio of 3:1. Women have more frequent relapses in the relapsing-remitting form (RRMS), while men have a faster onset of disability and initial relapse [1].

Two hypotheses of the etiology of MS have been proposed. The first is the autoimmune “outside-in” hypothesis, according to which unregulated autoreactive T-lymphocytes in the periphery penetrate the blood-brain barrier (BBB) and induce demyelination in the CNS together with macrophages and B-cells, which damages neuronal cells. The “inside-out” hypothesis is based on the assumption that the initial failure occurs within the CNS, as in other neurodegenerative diseases. MS should therefore be accompanied by varying degrees of inflammation. In predisposed individuals, the inflammatory reaction is promoted by the secretion of autoantigens and is therefore an autoimmune inflammatory reaction. According to this hypothesis, primary degeneration is present from the beginning several years before the first overt clinical symptoms and progresses throughout the course of the disease. Nonetheless, in both hypotheses, inflammation and neurodegeneration are present from the onset of the disease (see review [2]).

### 1.1. Multiple Sclerosis and Steroids

Sex hormones such as testosterone (T) and progesterone (P) are anti-inflammatory, while estradiol (E2) has a bipotential effect: pro-inflammatory at low concentrations and anti-inflammatory at high concentrations [3]. Changes in sex hormone levels are associated with exacerbation of MS just before the menstrual cycle, when E2 and P levels are very low [4]. Furthermore, the recurrence of MS symptoms decreases during the last three months of pregnancy and increases again after delivery, and these changes also seem to be related to immunological and hormonal changes [5].

#### 1.1.1. Cortisol

Cortisol is a steroid hormone produced by the *zona fasciculata* of the adrenal cortex. Cortisol increases the overall readiness of the body in stressful situations by increasing blood sugar through gluconeogenesis. Cortisol exhibits profound diurnal variations, with its levels peaking within 30–40 min after waking and subsequently declining across the rest of the day. Its decline is rapid in the morning and slows down in the afternoon until it reaches its lowest concentration around the onset of sleep [6]. Cortisol has primarily wide-ranging effects through genomic mechanisms. The effects of cortisol are mediated by its binding to two specific receptors, namely high-affinity mineralocorticoid receptors (MR) and low-affinity glucocorticoid receptors (GR) [6]. Its binding to these receptors is exercised at the onset of stress during its basal secretion. When cortisol levels rise during the stress response and at the peak of its circadian rhythm, its association with low-affinity GR gradually increases [7]. However, cortisol also has non-genomic effects, which are mediated by membrane receptors and G-protein-coupled receptor signaling [6].

In addition, cortisol affects the activity of several neurotransmitter systems that influence reward processing, attention regulation, executive function, mood, and emotion, such as type A GABA receptors (GABA_A_Rs), glutamate, dopamine, and acetylcholine receptors. Furthermore, cortisol suppresses the synthesis, release, and metabolism of serotonin, which increases the risk of depression [6] and also influences the release of excitatory amino acids and the induction of endocannabinoid synthesis [6]. In the context of stress and fear, cortisol affects a number of cognitive domains, including attention, perception, memory, and emotional processing. Increased cortisol secretion can lead to cognitive impairment [8,9,10].

#### 1.1.2. Δ^5^ Steroids

The adrenal *zona fasciculata* produces all the cortisol circulating in the body, while the adrenal *zona reticularis* produces approximately 80% of dehydroepiandrosterone (DHEA). The testes, ovaries, and brain synthesize the rest [6]. Unlike the nuclear receptors that bind cortisol, no specific receptor for dehydroepiandrosterone sulfate (DHEAS) has yet been identified [6]. While excess cortisol inhibits its own secretion through feedback mechanisms, these mechanisms are absent in DHEA and DHEAS, although adrenocorticotropic hormone (ACTH) stimulates both cortisol and DHEA/S synthesis in the adrenal glands [6]. Whereas long-term hypercortisolemia leads to cognitive impairments, DHEAS exerts anti-glucocorticoid activity, protecting the hippocampus from the harmful effects of cortisol [10,11,12].

Pregnenolone (Preg) formation is catalyzed by cholesterol desmolase, and this process is a rate-limiting step of adrenal steroidogenesis [6]. Preg, DHEA, Preg sulfate (PregS) and DHEAS cross the BBB and their adrenal production and/or therapeutic supplementation may affect their concentrations in the central nervous system (CNS) [13,14].

Preg, similarly to DHEA/DHEAS, has neuroprotective effects against glutamate-induced neurotoxicity, stabilizes microtubules, activates neurite growth, and promotes myelination [15,16]. PregS and DHEAS are neuroactive steroids (NASs) that can modulate several types of ionotropic receptors, such as N-methyl-D-aspartate receptors (NMDARs), α-amino-3-hydroxy-5-methyl-4-isoxazolepropionic acid receptors (AMPARs), melastatin receptors (TRPM3s), short transient receptor potential receptors 5 (TRPC5s), or vanilloid receptors (TRPV1s), and may improve cognitive function and counteract pain transmission and fear (see [8,17]). The neuroprotective effects of DHEA/DHEAS can also be attributed to their modulating effects on GABA_A_Rs and the protection of mitochondria from intracellular Ca^2+^ overload [15]. DHEA may protect hippocampal cells from the harmful effects of cortisol [8]. DHEAS and PregS act as agonists, whereas P functions as an antagonist on σ_1_Rs, which are present in high densities in the brain, and σ_1_Rs may exhibit a potent modulating effect on excitatory neurotransmitter (glutamatergic and cholinergic) systems [15,18]. DHEA also acts as a direct agonist on estrogen receptor β (ERβ) and a weak antagonist on androgen receptors [6].

DHEA/DHEAS has anti-inflammatory effects outside the CNS, reducing the levels of interleukin 1 (IL-1), interleukin 4 (IL-4), and interleukin 6 (IL-6), interleukin 12 (IL-12), and tumor necrosis factor α (TNF-α) [16].

#### 1.1.3. Active Androgens

The incidence of autoimmune diseases is generally lower in men, which is probably related to higher levels of active androgens. Hence, androgens are considered positive players in the development and function of the innate immune response since they inhibit the adaptive immune system, thereby protecting to some extent against autoimmunity [1].

#### 1.1.4. Estradiol

E2 is the main female sex hormone. Its synthesis is not restricted to the ovaries, and it is synthesized to a significant extent in extragonadal tissues, especially in adipocytes, which express the enzymes required for its synthesis, such as aromatase, converting androstenedione (A) to estrone, and aldoketoreductase AKR1C3, converting inactive estrone to active E2 (see reviews [19,20], see also http://biogps.org/#goto=genereport&id=8644, accessed on 21 August 2024). While the sex hormones T and P are anti-inflammatory, E2 has a bipotential effect: pro-inflammatory at low concentrations and anti-inflammatory at high concentrations (see [2]).

#### 1.1.5. Progesterone and Its Metabolites

In relation to the experimental autoimmune encephalomyelitis (EAE) model (serving as an experimental animal model for the investigation of MS), P shows a variety of effects, ranging from increasing neuronal vulnerability to inactivity to improving disease when administered with E2. During pregnancy, when P levels are extremely elevated, there is a reduction in disease activity, and it is therefore thought that P may have protective effects on MS. In addition, P mediates a reduction in nitric oxide production and toll-like receptor expression by macrophages. However, apart from the above protective effects, P increases the vulnerability of neurons to apoptotic damage in EAE. (see review [21]).

#### 1.1.6. Previous Multi-Steroid Studies Focused on Circulating Steroids in Multiple Sclerosis

Foroughipour et al. investigated 16 relapsing remitting MS (RRMS) patients (16 female MS patients in follicular phase and 16 female MS patients in luteal phase) with a mean Expanded Disability Status Scale (EDSS) score of 2 (1–5.5), while 30 age-matched healthy subjects served as controls at each phase of the menstrual cycle (MC). All volunteers were aged between 15 and 35 years, had normal menstrual cycles, and had no history of contraceptive pills or hormone replacement therapy. The mean (SD) age of RRMS patients was 27.2 (4.6) years and that of healthy controls was 26.2 (4.1) years [22]. The authors followed the levels of P, T, E2, cortisol, DHEA-S, and DHEA.

Besides our 2015 study, we quantified 51 steroids and steroid polar conjugates in the serum of 12 women with MS, untreated with steroids, and only 6 age-corresponding female controls with the use of gas chromatography—mass spectrometry (GC-MS) [23]. The results of this study differed from our present data, possibly due to the very low number of controls (n = 6) and different methods for steroid analysis [24].

Another multi-steroid study is from Caruso et al. [25], who quantified the levels of several steroids, such as Preg, P, 5α-dihydroprogesterone (5α-DHP), 3α,5α-THP, 3β,5α-THP, DHEA, T, 5α-dihydrotestosterone (5α-DHT), 3α,5α,17β-AD, and E2 by liquid chromatography-tandem mass spectrometry (LC-MS/MS) in plasma and CSF of 26 male adult MS patients and in 12 male controls.

## 2. Results

### 2.1. Alterations in Steroid Levels and Their Correlations with the Severity of MS

To assess the prevailing trend in the differences between patients and controls in steroid levels or steroid molar ratios, the Wilcoxon one-sample test with correction for continuity was used, and the following was used to record the data in the text: (number of data with significant increases in levels or values (*p* < 0.05)/number of data with no significant differences in levels or values/number of data with significant decreases in levels or values, statistical significance of the Wilcoxon test). Similarly, to assess the prevailing trend of correlations between steroid or steroid molar ratios and indicators of disease severity prior to treatment, the data entry in the text was as follows: (number of significant positive correlations (*p* < 0.05)/number of non-significant correlations/number of significant positive correlations, statistical significance of Wilcoxon test).

For a total of 81 steroids examined, there was a significant trend towards lower levels in patients compared to controls (5/61/15, *p* = 0.026). Regarding non-conjugated steroids, a highly significant trend towards lower values was observed (3/29/17, *p* = 0.008).

When evaluating the correlations of total steroids with EDSS, timed 25-foot walk (T25FWT), and 9-Hole Peg Test for the right hand (HPT9_R) and for the left hand (HPT9_L) in patients, there were significant trends towards positive correlations (12/69/0, *p* < 0.001), (19/62/0, *p* < 0.001), (24/57/0, *p* < 0.001), and (17/64/0, *p* < 0.001), respectively.

For the unconjugated steroids, there was a significant trend towards lower levels in patients (3/29/14, *p* = 0.008). The correlations of EDSS, T25FWT, HPT9_R, and HPT9 with unconjugated steroids showed significant trends towards positive correlations in patients (4/42/0, *p* = 0.047), (8/38/0, *p* = 0.005), (5/41/0, *p* = 0.026), and (4/42/0, *p* = 0.047), respectively.

For the conjugated steroids, no significant trend related to MS was found (2/32/1, *p* = 0.575). When evaluating the correlations of conjugated steroids with EDSS, T25FWT, HPT9_R, and HPT9 in patients, there were significant trends towards positive correlations (8/27/0, *p* = 0.005), (11/24/0, *p* < 0.001), (19/16/0, *p* < 0.001), and (13/22/0, *p* < 0.001), respectively.

#### 2.1.1. Corticoids (C21 Δ^4^ Steroids) and 11β-Hydroxy-androstanes (C19 Δ^4^ and 5α/β Steroids) and Their Correlations with the Grade of MS

Corticoids and 11β-hydroxy-androstanes did not show a significant overall trend in relation to MS (2/7/1, *p* = 0.608). While cortisol and cortisone levels were higher in patients than in controls, 11β-hydroxy-androsterone (11β-OH-3α,5α-THA) levels were lower here. The associations of corticoids and 11β-hydroxy-androstanes with EDSS, T25FWT, HPT9_R, and HPT9_R in patients tended towards positive correlations (4/7/0, *p* = 0.051), (5/6/0, *p* = 0.029), (5/6/0, *p* = 0.029), and (5/6/0, *p* = 0.029), respectively.

#### 2.1.2. Δ^5^ and Δ^4^ Steroids and Their Correlations with the Grade of MS

For the Δ^5^ and Δ^4^ steroids, no significant trend related to MS was found (2/21/7, *p* = 0.098). When evaluating the correlations of Δ^4^ and Δ^5^ steroids with EDSS in patients, no significant trend was found. When evaluating the correlations of Δ^4^ and Δ^5^ steroids with EDSS and T25FWT and HPT9_L in patients, no significant trend was found (2/28/0, *p* = 0.164), (3/27/0, *p* = 0.087), and (3/27/0, *p* = 0.087), respectively, while the associations of Δ^4^ and Δ^5^ steroids with HPT9_L tended towards positive correlations (5/25/0, *p* = 0.026).

#### 2.1.3. Active Androgens and Androstenedione

A and T levels were lower in patients compared to controls, while levels of unconjugated and conjugated 5α-DHT were not significantly different between controls and patients. No significant correlations were found for active androgens and androstenedione with indices of MS severity.

#### 2.1.4. Estradiol and Its Precursors

The levels of active female sex hormone E2 were significantly lower in patients compared to controls, like the levels of its precursors A as values of the ratios of E2/A and E2/T that may reflect the functioning of aromatase (CYP19A1). E2 levels, as well as E2/A, E2/T, and E2/(A+T) ratios, were not significantly correlated with EDSS, T25FWT, and HPT9_L, but positive correlations were found between HPT9_R on the one hand and E2/A and E2/(A+T) on the other.

#### 2.1.5. Progesterone and Its Metabolites

The levels of P and its metabolites showed a borderline tendency towards lower values in patients (1/27/7, *p* = 0.051).

The associations of progesterone and its metabolites with EDSS, T25FWT, and HPT9_R in patients tended towards positive correlations (6/29/0, *p* = 0.051), (9/26/0, *p* = 0.003), and (6/29/0, *p* = 0.015), respectively, while the associations of progesterone and its metabolites with HPT9_L did not show a significant trend (1/34/0, *p* = 0.331).

### 2.2. Alterations in Steroid Molar Ratios

#### 2.2.1. Steroid Sulfotransferase 2A1 (SULT2A1) vs. Steroid Sulfatase (STS)

As was already shown, the unconjugated steroids showed a highly significant trend towards lower values in patients, while the conjugated steroids were not significantly related to MS. Furthermore, when comparing the number of molar ratios that may reflect the balance between conjugated and unconjugated steroids, there was a significant trend towards higher values in patients (7/21/1, *p* = 0.035). When evaluating the correlations of molar ratios that may reflect the correlation of the balance between conjugated and unconjugated steroids with EDSS, T25FWT, and HPT9_R in patients, no significant trend was found (4/24/1, *p* = 0.185), (1/23/5, *p* = 0.106), and (6/21/2 *p* = 0.161), respectively. However, the association with HPT9_L showed a significant trend towards positive correlations (6/23/0, *p* = 0.015).

#### 2.2.2. C17-Hydroxylase, C17-20-Lyase (CYP17A1), Hydroxylase + Lyase Steps

The molar ratios that may reflect the functioning of CYP17A1 (hydroxylase + lyase steps) showed a significant trend towards higher values in patients (11/11/1, *p* = 0.004). Of the Δ^5^ and Δ^4^ steroids, only the DHEA/20α-DHPreg ratio was higher in patients than in controls, while the remaining molar ratios showing higher values in patients included 5α/β-reduced steroids. When evaluating the associations of molar ratios that may reflect the activity of CYP17A1 in both hydroxylase and lyase steps with EDSS and T25FWT, in patients, there were significant trends towards negative correlations (0/16/7, *p* = 0.009) and (0/17/6, *p* = 0.015), respectively. Alternatively, the associations of molar ratios that may reflect the activity of CYP17A1 in both hydroxylase and lyase steps with HPT9_L in patients tended towards positive correlations (5/18/0, *p* = 0.027); no significant trend was found for HPT9_R (3/19/1, *p* = 0.329).

#### 2.2.3. C17-Hydroxylase, C17-20-Lyase (CYP17A1), Hydroxylase Step

The molar ratios that may reflect the functioning of CYP17A1 in the hydroxylase step showed no significant trend related to MS was found (1/10/4, *p* = 0.191).

Also, the associations of molar ratios that may reflect the activity of CYP17A1 in the hydroxylase step with EDSS, T25FWT, HPT9_R, and HPT9_L showed no significant trends (0/15/0, *p* = 1), (3/10/2, *p* = 0.68), (0/15/0, *p* = 1), and (1/14/0, *p* = 1), respectively.

#### 2.2.4. C17-Hydroxylase, C17-20-Lyase (CYP17A1), Lyase Step

The molar ratios that may reflect the functioning of CYP17A1 in the lyase step did not show a significant trend related to MS (3/5/6, *p* = 0.334). When assessing the correlations of the above indices with EDSS, T25FWT, and HPT9_L in patients, there was a borderline or significant trend towards positive correlations (4/10/0, *p* = 0.05), (3/11/0, *p* = 0.091), and (3/11/0, *p* = 0.091), but this trend was completely absent in HPT9_R (0/14/0, *p* = 1).

#### 2.2.5. 3β-Hydroxysteroid Dehydrogenases (HSD3B1 and 2)

The molar ratios that may reflect the functioning of HSD3Bs showed a significant trend towards lower values in patients (0/5/6, *p* = 0.016). When evaluating the correlations of molar ratios that may reflect the activity of HSD3B with EDSS, T25FWT, HPT9_R, and HPT9_L in patients, no significant trend was found (1/10/0, *p* = 0.363), (0/11/0, *p* = 1), (3/7/1, *p* = 0.343), and (3/8/0, *p* = 0.094), respectively.

#### 2.2.6. 11β-Hydroxylase (CYP11B1)

The molar ratios that may reflect the functioning of CYP11B1 showed no significant trend related to MS, possibly due to the low number of events (0/4/3, *p* = 0.102). When evaluating the correlations of molar ratios that may reflect the activity of CYP11B1 with EDSS and T25FWT in patients, there was a significant trend towards positive correlations in patients (5/2/0, *p* = 0.032 for both indices). However, regarding the correlations with HPT9_R and HPT9_L, no significant trend was found (2/5/0, *p* = 0.192 for both indices).

#### 2.2.7. 11β-Hydroxysteroid Dehydrogenase, Type 1 (HSD11B1)

The molar ratios that may reflect the functioning of HSD11B1 whose values were significantly lower (n = 0), showed no significant change (n = 1), and were significantly higher in patients (n = 2) (*p* = 0.258 Wilcoxon’s test); no significant trend related to MS was found, possibly due to the low number of events (2/1/0, *p* = 0.258). When evaluating the correlations of molar ratios that may reflect the activity of HSD11B1 with EDSS, T25FWT, HPT9_R, and HPT9_L in patients, no significant trend was found (2/2/0, *p* = 0.225), (1/3/0, *p* = 0.453), (0/4/0, *p* = 1), and (1/3/0, *p* = 0.453), respectively.

#### 2.2.8. 7α-, 7β-, and 16α-Hydroxylating Enzymes (CYP7B1, CYP3A4, CYP3A7)

The 7α/β and 16α-hydroxy-steroids showed an insignificant trend to lower levels in patients (0/5/3, *p* = 0.099), probably due to the low number of records. Also, the molar ratios that may reflect the functioning of CYP7B1, 3A4, and 7 enzymes showed an insignificant trend to lower values in patients, probably due to the low number of records (1/2/5, *p* = 0.119).

When evaluating the correlations of molar ratios that may reflect the activities of CYP7B1, CYP3A4, and CYP3A4 with EDSS and HPT9_R in patients, significant trends towards positive correlations were observed (6/2/0, *p* = 0.018) and (5/3/0, *p* = 0.031), respectively. Concerning the correlations with T25FWT and HPT9_L, there were insignificant trends to positive correlations (4/4/0, *p* = 0.054 for both indices).

The molar ratios, which may primarily reflect the 7α/β-hydroxylation activity for Δ^5^ androgens catalyzed, namely by CYP7B1, showed an insignificant trend toward lower values in patients, likely due to the low number of events (4/0/0, *p* = 0.072).

When assessing correlations of molar ratios, which may reflect 7α/β-hydroxylation activity for Δ^5^ androgens, with EDSS, T25FWT, HPT9_R, and HPT9_L, insignificant tendencies towards positive correlations were found in patients (4/0/0, *p* = 0.072 for all quoted indices).

#### 2.2.9. 5α-Reductase (SRD5A1, SRD5A2)

The levels of 5α-reduced steroids did not show a significant trend related to MS (2/25/4, *p* = 0.422). However, when evaluating the associations of 5α-reduced steroids with EDSS, T25FWT, HPT9_R, and HPT9_L in patients, there was a significant trend towards positive correlations (7/24/0, *p* = 0.008), (14/17/0, *p* < 0.001), (11/20/0, *p* < 0.001), (8/23/0, *p* = 0.005), respectively. The molar ratios that may reflect the functioning of SRD5As showed no significant trend related to MS was found (1/11/1, *p* = 1). When evaluating the associations of molar ratios that may reflect the activities of SRD5As with EDSS, T25FWT, and HPT9_R in patients, insignificant or borderline trends towards positive correlations were found (3/10/0, *p* = 0.092), (4/9/0, *p* = 0.05), and (3/10/0, *p* = 0.092), respectively, but the absence of a significant trend was observed for HPT9_L (2/11/0, *p* = 0.175).

#### 2.2.10. 5β-Reductase (AKR1D1)

Our data show that there is no significant trend in the differences between patients and controls for 5β-reduced steroids (1/15/3, *p* = 0.175). When evaluating the associations of 5β-reduced steroids with EDSS and T25FWT in patients, no significant trend was found (3/16/0, *p* = 0.089) and (2/17/0, *p* = 0.169), respectively. Alternatively, for HPT9_R and HPT9_L, there were significant trends towards positive correlations (8/11/0, *p* = 0.005) and (6/13/0, *p* = 0.015), respectively.

Molar ratios, which may reflect AKR1D1 functioning, did not show a significant trend associated with MS (2/11/0, *p* = 0.175), nor did the associations of these molar ratios with EDSS, T25FWT, HPT9_R, and HPT9_L in patients (0/13/0, *p* = 1), (0/13/0, *p* = 1), (2/11/0, *p* = 0.175), and (2/11/0, *p* = 0.175), respectively.

#### 2.2.11. Aldoketoreductase 1C1 (AKR1C1) vs. 17β-Hydroxysteroid Dehydrogenase, Type 2 (HSD17B2)

The molar ratios that may reflect the balance between AKR1C1 and HSD17B2 of AKR1C1 did not show a significant trend related to MS (4/12/2, *p* = 0.429). These molar ratios also did not show significant trends in correlations with EDSS, T25FWT, HPT9_R, and HPT9_L in patients (0/18/0, *p* = 1), (1/15/2, *p* = 0.587), (0/17/1, *p* = 0.345), and (0/17/1, *p* = 0.345), respectively.

#### 2.2.12. Aldoketoreductase 1C2 (AKR1C2) vs. 17β-Hydroxysteroid dehydrogenase, Type 2 and 6 (HSD17B2, HSD17B6)

The molar ratios that may reflect the balance between AKR1C2 on the one hand and HSD17B2 and HSD17B6 on the other did not show a significant trend related to MS (3/13/7, *p* = 0.212). However, the associations of these molar ratios with EDSS in patients showed a trend towards negative correlations (0/16/7, *p* = 0.009), and these associations with T25FWT showed a borderline tendency towards negative correlations as well (1/16/6, *p* = 0.061). Alternatively, there was no significant trend for HPT9_R and HPT9_L (1/19/3, *p* = 0.329) and (1/21/1, *p* = 1), respectively.

The molar ratios of 3α-hydroxy to 3β-hydroxy 5α/β-reduced steroids showed a significant trend towards lower values in patients (0/9/7, *p* = 0.009). When evaluating associations of these molar ratios with EDSS in patients, there was a significant trend towards negative correlations (0/12/4, *p* = 0.049). However, for T25FWT, HPT9_R, and HPT9_L, there was no significant trend in the aforementioned associations (1/12/3, *p* = 0.334), (1/13/2, *p* = 0.59), and (0/15/1, *p* = 0.349), respectively.

The molar ratios of 3α- to 3-oxo- 5α/β-reduced steroids showed no significant trend related to MS, possibly due to the low number of events (0/4/3, *p* = 0.102).

The associations of the aforementioned molar ratios with EDSS and T25FWT in patients did not show significant trends, possibly due to the low number of records (0/4/3, *p* = 0.102 for both indices), and this trend has faded for HPT9_R and HPT9_L (0/6/1, (*p* = 0.391 for both indices).

#### 2.2.13. Aldoketoreductase 1C3 (AKR1C3) vs. 17β-Hydroxysteroid Dehydrogenase, Type 2 (HSD17B2)

The molar ratios that may reflect the balance between AKR1C3 and HSD17B2 showed a borderline trend towards higher values in patients (4/9/0, *p* = 0.05). However, when evaluating associations of these molar ratios with EDSS and T25FWT in patients, there was a borderline trend towards negative correlations (*p* = 0.05, Wilcoxon’s test) (0/9/4, *p* = 0.05), and this trend has faded for HPT9_R and HPT9_L (0/12/1, *p* = 0.356) and (1/12/0, *p* = 0.356), respectively.

## 3. Discussion

### 3.1. Altered Steroid Levels in Patients and Their Correlations with the Severity of MS

Our present data show significant steroidomic changes in patients before treatment, suggesting an important role for steroids in the pathophysiology of MS and the potential to exploit these differences in its diagnosis. The aggregated results show a significant trend towards lower steroid levels in patients. Since, with the exception of mid-cycle female sex hormones and the luteal menstrual phase, most steroids in women are synthesized either directly in the adrenal glands or from adrenal precursors [26] these results suggest an overall trend toward impaired adrenal activity in patients. This trend may affect the synthesis of other steroids (including active steroid hormones and neuroactive, neuroprotective, and immunoprotective steroids) found downstream in the metabolic pathway.

Surprisingly, in contrast to the trend towards lower steroid levels in patients compared to controls, a trend towards positive correlations of steroid levels with the MS severity was found. This discordance could mean that although lower steroid levels could be related to the onset of the disease, there could be a counter-regulation towards an increase during the progression of the disease. In addition, lower steroid levels could be an indicator of a predisposition to MS. Conversely, during the development of MS, the differences between steroid levels in patients and controls should diminish, which could hinder the differentiation of patients with more serious MS from controls.

#### 3.1.1. Corticoids (C21 Δ^4^ Steroids) and 11β-Hydroxy-androgens (C19 Δ^4^ and 5α/β Steroids)

MS is an autoimmune disease induced by autoreactive T-lymphocytes that is characterized by an imbalance of pro-inflammatory cytokines, such as TNF-α, IFN-γ, IL-2 and lymphotoxin, and regulatory cytokines (e.g., IL-4 and IL-10). Cytokines stimulate the pituitary gland via the hypothalamus to produce adrenocorticotropic hormone, which stimulates cortisol production in the *zona fasciculata* [27,28,29]. Glucocorticoid production is regulated by hypothalamic corticotropin-releasing hormone (CRH) and pituitary ACTH, but also by cytokines such as IL-1, IL-6, and TNF-α. The bioavailability of cortisol also depends on its interconversion to cortisone, which is inactive, and the balance between bioactive cortisol and inactive cortisone is regulated by the reductive enzyme HSD11B1 (in the direction of cortisol) and the oxidative enzyme HSD11B2 (in the direction of cortisone). Glucocorticoids play a decisive role in the regulation of the immune system and act through binding to the GR. Although glucocorticoids are mainly a product of the adrenal *zona fasciculata*, they can be produced extra-adrenally, for example in cells of the immune system, intestine, skin, or brain [30].

HPAA, which also controls the cortisol response to emotional and cognitive stress [31,32], also regulates the interplay between peripheral inflammatory processes and cortisol production. High levels of cortisol inhibit the activity of the hypothalamic paraventricular nucleus, which produces CRH, via the brainstem, creating a negative feedback mechanism [28,29,33]. The hypothalamic-pituitary-adrenal axis (HPAA) is overactive in MS patients, and many studies have reported elevated cortisol levels in MS patients regardless of the type of body fluid in which cortisol was measured (summarized in [34]).

Some studies have reported unaltered circulating cortisol levels in patients or even reduced cortisol levels in the cerebrospinal fluid of patients compared with controls [22,35]. In spite of unaltered cortisol levels in relation to MS found in their study, Foroughipour et al. [22] hypothesized that chronically activated HPAA in female patients [36], peripheral gonadotropin resistance in combination with abnormal central regulation stimulates increased pituitary follicle-stimulating hormone (FSH) secretion [37]. Also, Wei and Lightman observed intact HPAA in the majority of MS patients and suggested that HPAA is unlikely to play a major role in the initial pathogenesis of MS [38]. The authors also suggested that overactivation of the HPAA in MS patients is secondary to an active inflammatory stimulus [38].

Consistent with the studies reporting elevated cortisol levels in MS patients, our current data also show elevated levels of the active glucocorticoid cortisol and its inactive metabolite cortisone in patients but unaltered levels of corticosterone, which is a precursor on the pathway to mineralocorticoid aldosterone. Except for lower levels of 11β-OH-3α,5α-THA in patients, the levels of remaining 11β-hydroxy-androstanes were unaltered, which may indicate unaltered functioning of CYP11B1 in MS patients.

#### 3.1.2. Δ^5^ and Δ^4^ Steroids

Aggregate results for Δ^5^ and Δ^4^ steroids showed a nonsignificant trend toward lower levels in patients than in controls. On the one hand, these results may indicate attenuated functioning of the adrenal cortex, but on the other, the absence of serious blocks in the pathway to cortisol synthesis, which is indicated by lower levels of 17-OH-P (the penultimate precursor on the pathway to cortisol) at elevated levels of cortisol and cortisone.

Caruso et al. [25] found higher levels of Preg in male MS patients in both plasma and CSF, compared to controls, whereas in our present data in women we did not observe a significant difference.

In the study by Foroughipour et al. [22], DHEA-S levels were significantly lower in both follicular and luteal phases in RRMS patients of reproductive age, whereas no significant difference between patients and controls was found in the levels of DHEA [22]. In contrast, our present data showed no changes in the levels of nine Δ^5^ androstanes including DHEA and DHEA, and only the levels of 7β-hydroxy-DHEA (7β-OH-DHEA) were decreased. It should be noted that 7β-OH-DHEA belongs to a group of immunoprotective Δ^5^ androstanes that alleviate the severity of autoimmune diseases [39,40,41,42,43,44]. Caruso et al. [25] also found no difference in DHEA in male MS patients compared to controls, but Noorbakhsh et al. reported that although levels of the main precursor of NASs, pregnenolone, did not differ between clinical groups, there was a significant reduction in DHEA levels in the white matter of patients compared to controls [45].

#### 3.1.3. Active Androgens and Androstenedione

While A alone does not appear to be a key bioactive steroid directly involved in the pathophysiology of MS, active androgens such as A metabolite T and T metabolite 5α-DHT play an important modulatory role but probably with different effects in women and men (see review [21,46]). T is directly (via biding on androgen receptors) and indirectly (through conversion to estrogens) involved in the modulation of the mesocorticolimbic system and affects the density of dopaminergic neurons expressing tyrosine hydroxylase participating in dopamine synthesis [47].

EAE is usually induced by helper T-lymphocytes (Th1 and Th17) and is characterized by the presence of characteristic cytokines such as IFN-γ and IL-17 in the brain, secondary lymphoid organs, and circulation. Both MS patients and animal models of EAE show a predilection for Th1, and this phenotype may be associated with low T levels (see review [46]), consistent with our current data. However, only T, not 5α-DHT, has a direct neuroprotective effect, suggesting that T and 5α-DHT may have independent effects on hippocampal and infiltrating immune cells (see review [46]).

Interestingly, in patients, higher T concentrations were significantly associated with the likelihood of irreversible tissue damage [36]. Given the relationship between serum sex hormone levels and MRI results, it is reasonable to assume that the increase in E2 and T levels in RRMS could be related to the tissue response to brain damage [36]. Although there were no significant correlations of active androgens and androstenedione with indicators of disease severity, this finding is consistent with our explanation for the discrepancy between the trend towards generally lower steroid levels in MS patients and the trend towards positive correlations between steroid levels and MS severity (before treatment).

In general, androgens provide a shift from a Th1 to a Th2 phenotype based on increased production of IL-5 and IL-10 and decreased pro-inflammatory cytokines, including IFN-γ, TNF-α, and IL-17. In this context, it should be noted that increased IFN-γ secretion may contribute to the known susceptibility of female experimental animals to the induction of autoimmune diseases, including EAE. In addition to active androgens, also their precursors suppress EAE [1].

Furthermore, T suppresses the proliferation and differentiation of lymphocytes and may inhibit the production of immunoglobulins. T can cross the BBB and directly interact with neuronal cells, thereby protecting neuronal cells from glutamate toxicity, increasing neurite outgrowth, protecting neuronal cell lines from oxidative stress, and increasing BDNF expression [1]. Androgen treatment also indirectly leads to an increase in thymocyte (T-cell) apoptosis, which could be another mechanism by which T could be protective in EAE (see review [21]). In addition, T reduces reactive gliosis and astrocyte proliferation, which are important aspects of axonal regeneration [48,49,50]. Moreover, T plays an important role in the repair of brain lesions, and the protective effects of this hormone have been demonstrated in both patients and animal models of MS [51].

On the one hand, sex hormones can protect brain tissue, but on the other hand, they can have excitotoxic and apoptotic effects. For example, T enhances excitotoxic damage to cultured oligodendrocytes, which may explain the worse prognosis of MS in men [52]. On the other hand, women with MS with low T levels had a higher number of enhancing lesions than women with MS with normal T levels (see review [21]).

In our present data, A and T levels were lower in patients compared to controls, while levels of unconjugated and conjugated 5α-DHT were not significantly different between controls and patients. Our results for T were consistent with those of Foroughipour et al. [22] and other authors also reporting lower T levels in female patients compared to controls (see review [46]).

**Figure 1:**
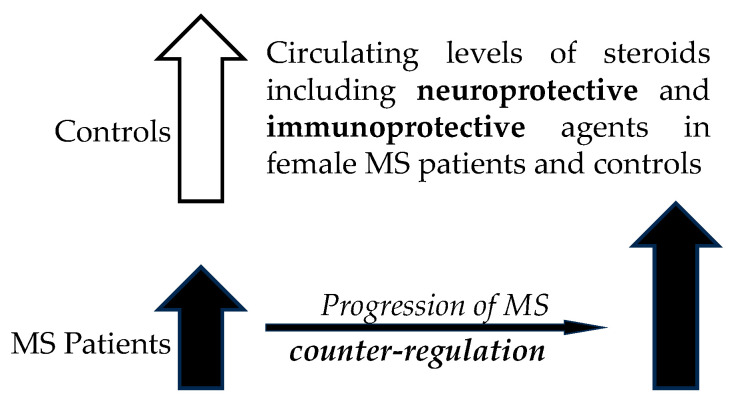
Comparison of circulating steroid levels in patients with early MS, advanced MS, and controls.

To summarize, our present data are consistent with data in the literature reporting lower levels of predominantly neuroprotective and immunoprotective T in patients (see review [46]). Furthermore, the aforementioned literature data are consistent with our explanation for the discrepancy between the trend towards generally lower steroid levels in MS patients and the trend towards positive correlations between steroid levels and MS severity. While low levels of steroids may be related to the pathogenesis of MS, the trend towards positive correlations between steroid levels and MS severity may be a part of the counter-regulatory mechanism against the disease progression (Figure 1).

#### 3.1.4. Estrogens, Their Precursors, and Aromatase Functioning

Estradiol is anti-inflammatory through inhibition of the production of pro-inflammatory cytokines (TNF-α, IL-1, and IL-6), inhibition of NK cell activation, and induction of anti-inflammatory cytokine expression (IL-4, IL-10), and promotes the Th2 phenotype with expression of transforming growth factor-β (TGF-β) and activation of T-reg cells [1].

Estradiol counteracts excitotoxicity via modulation of glutamate NMDARs and AMPARs density, oxidative stress, inflammation, or apoptosis, acting as a free radical scavenger. Estradiol may also enhance cognition by reducing inflammation and could also be neuroprotective by maintaining mitochondrial function and may maintain calcium concentrations, preventing cell death. In addition, E2 influences intracellular Ca^2+^ concentration and modulates Na+/K+-ATPase and membrane fluidity (see reviews [21,47,53,54,55]).

Transcriptional effects on enzyme production mediate the action of estrogens on the cholinergic, serotonergic, and glutamatergic systems. Experimental modeling of MS shows that estrogens, via binding to ERα, counteract disease activity (see review [21]).

Moreover, estrogens regulate the CNS dopaminergic system by affecting the expression and function of dopamine receptors and transporters. The neurotransmitter dopamine mediates neuropsychological functions of the CNS and also modulates cells of the innate and adaptive immune system, including Th17 cells, which play a key role in inflammatory CNS diseases including MS; see review [56]. Dopamine is also implicated in depression, cognitive impairment, and fatigue in MS, and depression is one of the main symptoms of MS and can exacerbate its severity. In addition, dopamine modulates the gut-brain axis, which is critical in neuroinflammation, autoimmunity, and psychiatric disorders [56]. It should also be noted that E2 negatively correlates (r = −0.471; *p* = 0.008) with EDSS scores [22]. E2 also reduces reactive gliosis and astrocyte proliferation, which are important aspects of axonal regeneration [48,49,50].

Our present data show that the levels of active female sex hormone E2 were significantly lower in patients compared to controls, like the levels of its precursors A and T. The E2/A and E2/T ratios, which may reflect aromatase (CYP19A1) function, were also lower in patients. Thus, in light of the above literature data, our present data support the suggestions that reduced levels of estradiol and also suppressed functioning of CYP19A1 may be associated with the pathophysiology of MS. Our results are in accordance with the data by Foroughipour et al., who found significantly lower E2 levels in the follicular phase [22] as well as with the study by Trenova et al., reporting that 60% of patients had serum concentrations of estradiol and/or P below the lower limit of normal in one or both phases of MS [57], and may also be consistent with the trend towards lower levels of their precursors A and T found in this study and elsewhere (see also review [46]). E2 levels, as well as E2/A, E2/T, and E2/(A+T) ratios, were not significantly correlated with three indices of MS severity, but positive correlations were found between HPT9_R on the one hand and E2/A and E2/(A+T) on the other, which may be part of a counter-regulatory mechanism against MS progression (Figure 1).

#### 3.1.5. Progesterone and Its Metabolites

P has neuroprotective and promyelinating effects on the CNS. In the spinal cord, P increases protect cultured neurons from glutamate toxicity and normalize functional defects of injured neurons. P also enhances proliferation and differentiation of oligodendrocyte precursor cells, which play an important role in remyelination after toxin-induced lesions (see review [21]).

Besides its myelinating effects [58,59], P modulates the immune system and changes the pro-inflammatory Th1 response into an anti-inflammatory Th2 response favoring Treg cell differentiation and supporting the reduction of interferon γ (IFN-γ) production by NK cells and glucocorticoid-mediated thymocyte apoptosis (see review [21]).

Concerning P, we did not find a difference between patients and controls, which was consistent with the data by Foroughipour et al. [22]. However, Tomassini et al. reported slightly higher levels in patients in the follicular phase [36]. Caruso et al. [25] found higher levels of P in male MS patients in plasma compared to controls but not in CSF. Generally, P and its metabolites showed a significant trend towards lower values in patients compared to controls. Specifically, P metabolites with lower levels in patients included 17-OH-P, 16α-OH-P, and numerous 3α-hydroxy-5α/β-pregnanes (including the most potent GABAergic modulator 3α,5α-THP) that are or may be potentially neuroprotective. The 3α,5α-THP alleviates neurobehavioral deficits and reduces neuropathology and inflammation in animal models of autoimmune demyelination (a hallmark of MS) and has various neuroprotective effects [45,60].

To date, no study has compared circulating levels of 5α/β-reduced pregnancies in women with and without MS. Regarding the altered levels of 3α,5α-THP in our present data, our results are in agreement with those of Noorbakhsh et al., who found lower white matter levels of allopregnanolone and other steroids at autopsy in MS patients compared to age- and sex-matched controls [45]. Reduced expression of neurosteroidogenic enzymes, along with reduced levels of allopregnanolone, was also observed in the brains of mice with EAE [60]. Meanwhile, treatment with allopregnanolone in a mouse model of EAE alleviated related neuropathology, including neuroinflammation, myelin and axonal injury, and reduction of neurobehavioral deficits [45]. A multi-platform study by Noorbakhsh et al. reveals consistently disrupted neurosteroidogenesis in both MS and EAE [45]. Recent studies have also shown that 3α,5α-THP can block neuroinflammation through activation of TLR4 protein in immune cells (macrophages) and in the brain, thereby suppressing inflammation [61].

Interestingly, pregnancy (characterized by extremely high levels of progesterone and its neuroprotective metabolites, especially allopregnanolone and pregnanolone) in MS patients is associated with a lower risk of disease progression and a lower rate of disease exacerbation, but disease recurrence occurs after delivery. Since the pathogenesis of MS seems to involve cellular immune reactivity at the expense of cell-mediated immunity, the alleviation of MS in pregnancy could be related to a transient weakening of cell-mediated immunity during this period. Pregnancy leads to a shift towards a Th 2 cytokine profile that is likely protective for MS [62].

In line with the general tendency towards lower steroid levels and positive correlations between steroid levels and MS severity, the same pattern was also observed for progesterone and its metabolites, again leading to the consideration of a counter-regulatory mechanism preventing MS progression (Figure 1).

### 3.2. Altered Steroid Molar Ratios in Patients

#### 3.2.1. Steroid Sulfotransferase 2A1 (SULT2A1) vs. Steroid Sulfatase (STS)

Regarding NASs, unconjugated steroids and their corresponding sulfates often have opposing effects on the same receptors and/or may be antagonists in relation to neuronal activity [63,64,65]. In addition, the sulfates of Preg and DHEA modulate several types of ionotropic receptors, such as NMDARs, AMPARs, nicotinic, TRPM3s, TRPC5s, or TRPV1s, and may improve cognitive function while counteracting pain and fear transmission [8,17].

**Figure 2:**
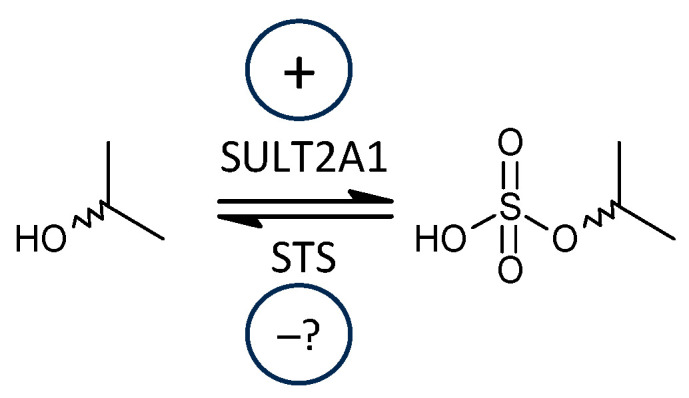
Scheme of the balance between steroid sulfotransferase 2A1 (SULT2A1) and steroid sulfatase (STS); the symbols +, ~, and −, represent higher, unaltered, and lower level or molar ratio, respectively.

Regarding our current data, the aggregated results showed a significant trend towards lower steroid levels in patients, but there was a clear difference between unconjugated and conjugated steroids. While the former group showed a highly significant trend towards higher values in patients than controls, the latter group did not show a significant trend, probably due to increased SULT2A1 activity in patients. Moreover, the molar ratios that may reflect the balance between SULT2A1 and STS show a significant trend towards elevated values in patients compared to controls, which may indicate an increased SULT2A1 activity in patients (Figure 2).

These results could be important in another aspect when it comes to the bioactivity of steroids, since steroids acting through binding to nuclear receptors are active in their unconjugated form, while their sulfates are inactive but can serve as a reservoir of substrates for conversion to active hormones [66]. In addition, sulfation of nonconjugated neuroprotective GABAergic steroids leads to their inactivation or formation of their antagonists [64,67], and conversely, some sulfated steroids may be positive or negative modulators of excitatory glutamate receptors, while all their nonconjugated analogues are inactive in this respect [68,69].

#### 3.2.2. C17-Hydroxylase, C17-20-Lyase (CYP17A1), Hydroxylase + Lyase Steps, and the Pathway to Cortisol Synthesis

**Figure 3:**
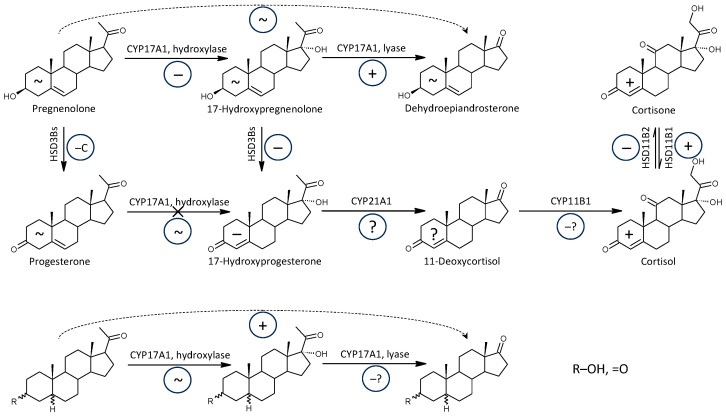
Simplified scheme of the functioning of C17-hydroxylase, C17,20 lyase in Δ5, Δ4, and “backdoor pathway” and a pathway to the biosynthesis of cortisol; the symbols +, ~, −, ? below the arrows showing steroid conversions represent higher, unaltered, lower, and unavailable/possibly altered level or molar ratio, respectively, while these symbols inside the steroid A–circle show the same for steroid levels; the symbol C denotes that the symbol—belongs to the molar ratio of progesterone to pregnenolone sulfate; the dashed arrow shows the overall trend in the conversion of 17-deoxy-pregnanes to androstanes.

The molar ratios that may reflect CYP17A1 (hydroxylase + lyase) functioning (without corticosteroids) were higher in patients, but of the Δ^5^ and Δ^4^ steroids, only the DHEA/20α-DHPreg ratio was elevated in patients compared to controls. Molar ratios showing higher values in patients included mainly 5α/β-reduced steroids, and this trend was highly significant (Figure 3).

#### 3.2.3. C17-Hydroxylase, C17-20-Lyase (CYP17A1), Hydroxylase Step, and the Pathway to Cortisol Synthesis

The molar ratios that may reflect the functioning of CYP17A1 in the hydroxylase step (without corticoids) showed no significant trend related to MS. However, the molar ratio values of 17-hydroxypregnenolone (17-OH-Preg) to Preg were lower in patients. The question is whether this finding may be related to specifically reduced CYP17A1 activity in the hydroxylase step in the Δ^5^ pathway, which is key in cortisol synthesis.

#### 3.2.4. C17-Hydroxylase, C17-20-Lyase (CYP17A1), Hydroxylase Step, and the Pathway to Cortisol Synthesis

The molar ratios that may reflect the functioning of CYP17A1 in the lyase step (without corticoids) showed no significant trend related to MS. However, the molar ratio values for Δ^5^ steroids and Δ^4^ steroids such as DHEA/17-OH-Preg and A/17-OH-P were higher in patients, while the molar ratios for 5α/β-reduced steroids insignificantly tended to lower levels in patients compared to controls, possibly due to the low number of events.

Given previous results, it is interesting that Gupta et al. showed a rapid conversion of 17-hydroxyallopregnanolone (3α,5α17-PD) to androsterone (3α,5α-THA) catalyzed by CYP17A1 in a lyase step, even in the absence of CYB5, and further reported that 3α,5α17-PD was a better substrate for CYP17A1 than 17-OH-Preg [70]. Although CYB5, which activates the CYP17A1 lyase step, has low tissue specificity, its expression in the adrenal cortex is about four times higher than in most other tissues (http://biogps.org/#goto=genereport&id=80777, accessed on 21 August 2024). In contrast, CYP17A1 is more than 1500 times more expressed in the adrenal cortex compared to most other tissues and about 70 and 40 times more expressed in the kidneys and testes, respectively (http://biogps.org/#goto=genereport&id=1586, accessed on 21 August 2024).

**Figure 4:**
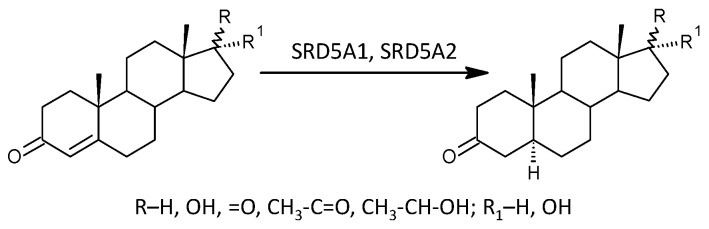
Scheme of the actions of 5α-reductases (SRD5A1 and SRD5A2).

While the formation of Δ^5^ steroids occurs mainly in the adrenal cortex, 5α/β-reduced steroids are mainly formed extra-adrenally. From the enzymes forming the 5α/β-reduced steroids, SRD5A1 is tissue non-specific (http://biogps.org/#goto=genereport&id=6715, accessed on 21 August 2024); SRD5A2 has low tissue specificity with about 3-fold higher expression in the liver compared to other tissues (http://biogps.org/#goto=genereport&id=6716, accessed on 21 August 2024). Both isoforms of SRD5A convert Δ^4^ steroids to 5α-reduced counterparts (Figure 4).

**Figure 5:**
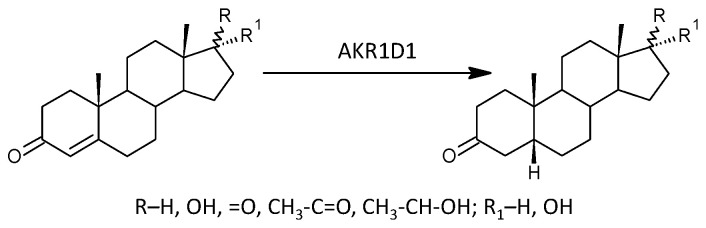
Scheme of the action of 5β-reductase (AKR1D1).

AKR1D1 converting Δ^4^ steroids to 5β-reduced counterparts is liver-specific, with about 60-fold higher expression in the liver compared to other tissues (http://biogps.org/#goto=genereport&id=6718, accessed on 21 August 2024) (Figure 5).

The above data indicate that extra-adrenal conversion to 5α/βreduced 17-deoxy-pregnanes to androstanes may be independent of the CYB5 enzyme, which is under-expressed outside the adrenal *zona reticularis* [71]. This may be the reason why, in the synthesis of Δ^5^ androgens (which make up the bulk of gonadal T precursors), the adrenal cortex prefers the Δ^5^ pathway, while other tissues, and especially the liver, prefer the synthesis of 5α/β-reduced androstanes independent of CYB5. Indeed, as our results showed, circulating levels of intermediates such as 17-hydroxyallopregnanolone sulfate (3α,5α,17-PDC), 3α,5β,17-PD, and 3α,5β,17-PD) on the pathway from 17-deoxy-5α/β-reduced pregnanes to 5α/β-reduced androstanes were disproportionately lower compared to the latter steroids, particularly the sulfated ones, indicating their very rapid conversion to the corresponding androstanes.

In summary, overall CYP17A1 functioning remained unchanged in patients on the Δ^5^ and Δ^4^ pathways, with attenuated CYP17A1 functioning in the hydroxylase step but enhanced CYP17A1 functioning in the lyase step, whereas overall CYP17A1 functioning was higher in the “backdoor” pathway. These results could have implications for the synthesis of and metabolism of bioactive steroids, be they cortisol, active androgens and estrogens, or neuroactive and neuroprotective substances acting through modulation of ionotropic receptors.

#### 3.2.5. 3β-Hydroxysteroid Dehydrogenases (HSD3B1 and 2)

**Figure 6:**
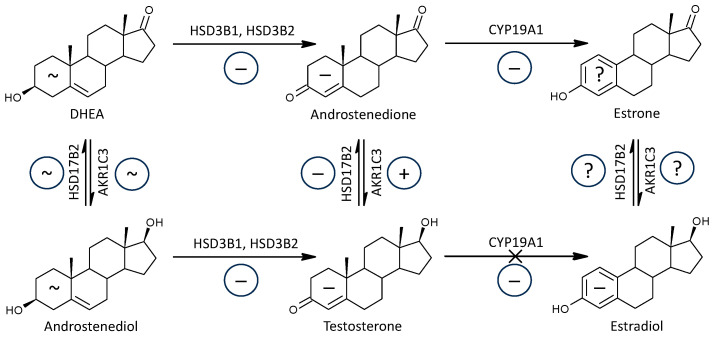
Simplified scheme of the alterations in the synthesis and metabolism of active androgens and estrogens; the symbols +, ~, −, ? represent higher, unaltered, lower, and unavailable/possibly altered steroid molar ratios, respectively, while these symbols inside the steroid A–circle show the same for steroid levels.

In our present data, molar ratios, which may reflect HSD3B functioning, showed a significant trend toward lower values in patients (Figure 3), although transcriptional analyses in the study by Noorbakhsh et al. showed no significant difference in HSD3B1 and HSD3B2 transcripts in white matter of patients and controls [45]. The expression of HSD3Bs isoforms in peripheral and brain may be different, and this inconsistency remains to be elucidated. The impaired conversion of Δ^5^ to Δ^4^ steroids in peripheral steroid-producing tissues may affect the pathways to cortisol as well as to active androgens and estrogens (Figure 6). However, this impairment does not preclude increased production of cortisol and corticosterone in the adrenal *zona fasciculata* related to excessive HPAA activation in patients (see review [34]).

#### 3.2.6. 11β-Hydroxylase (CYP11B1)

CYP11B1 catalyzes the final step in cortisol synthesis as it converts 11-deoxycortisol to cortisol (Figure 3) and simultaneously 11-deoxy-androstanes to 11β-hydroxy-androstanes. It should be pointed out that the 11β-hydroxy-androstanes cannot be formed from cortisol and its 5α/β-reduced metabolites under CYP17A1 catalysis in the lyase step [72].

Except for lower levels of 11β-OH-3α,5α-THA in patients, the levels of the remaining six 11β-hydroxy-androstanes were unaltered, which may indicate unaltered functioning of CYP11B1 in MS patients. However, the molar ratios that may reflect the functioning of CYP11B1 indicated an insignificant trend (possibly due to the low number of events) to their lower values in patients compared to controls. Therefore, the last metabolic step in cortisol synthesis could also be disrupted in patients. However, even this disruption does not prevent the resulting increased production of cortisol and cortisone (Figure 3).

While EDSS and T25FWT in patients showed significant trends towards positive correlations with molar ratios that may reflect the activity of CYP11B1, these trends were absent for HPT9_R and HPT9_L. This result could also be related to a counter-regulatory mechanism shifting steroidogenesis towards a situation common in controls at higher MS severity (Figure 1).

#### 3.2.7. 11β-Hydroxysteroid Dehydrogenase, Type 1 (HSD11B1)

HSD11B1 is an important diabetogenic enzyme that converts inactive cortisone to the active glucocorticoid cortisol [73]. Briefly sketching the scheme of cortisol synthesis and metabolism, it can be illustrated as follows: Preg -> 17-OH-Preg (CYP17A1, hydroxylase) -> 17-OH-P (HSD3B2) -> 11-deoxycortisol (CYP21A2, 21-hydroxylase) -> cortisol (CYP11B1) <=> cortisone (HSD11B1 vs. HSD11B2) (Figure 3).

**Figure 7:**
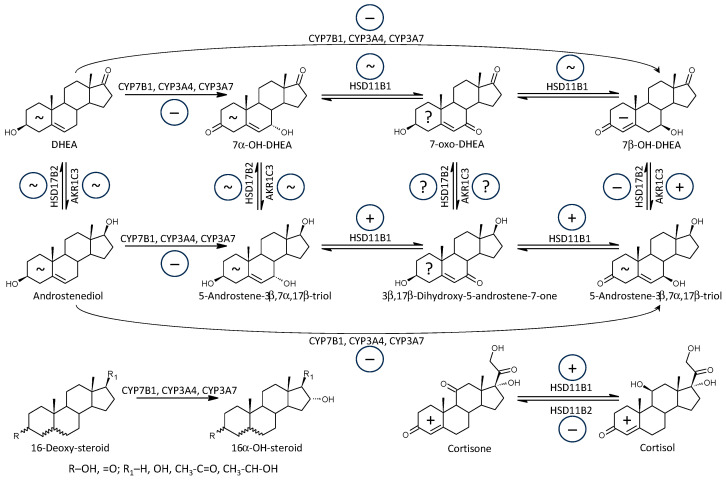
Simplified scheme of the synthesis and interconversion of 7α-, 7β-, and 16α-hydroxy-Δ^5^-androstanes and the interconversion of cortisol and cortisone; the symbols +, ~, −, ? represent higher, unaltered, lower, and unavailable/possibly altered steroid molar ratios respectively, while these symbols inside the steroid A–circle show the same for steroid levels.

Although of the 3 molar ratios that may reflect HSD11B1 functioning, two showed elevated values in patients, including the cortisol to cortisone molar ratio, which could indicate a shift from HSD11B2 (which converts cortisol to inactive cortisone) to the antagonistic HSD11B1, which acts in the opposite way. Nevertheless, besides cortisol, the cortisone values were higher in patients. HSD11B1 and HSD11B2 have distinct tissue expression patterns and contribute differently to circulating and local cortisol levels. While HSD11B1 is more widespread and mainly involved in cortisol activation, HSD11B2 focuses on cortisol inactivation in specific tissues. The enzyme is localized in selective tissues so that it can act as a paracrine or autocrine protector of the receptor against the action of the active form of glucocorticoid [74]. The above data suggest that HSD11B1 may be more important for the balance between cortisol and cortisone in the circulation than HSD11B2 [75]. Steroid interconversion involving HSD11B1 functioning is shown in Figure 7 and demonstrates a shift in the balance between HSD11B1 and HSD112B2 functioning in favor of the former isoform.

Suppression of catabolism of bioactive cortisol to inactive cortisone could partially compensate for bottlenecks in the metabolic pathway to bioactive cortisol, such as impaired HSD3B2 and CYP11B1 function, as well as increased conversion of 17-OH-PREG to DHEA (metabolizing 17-OH-PREG, which is one of the precursors of cortisol). It can be speculated if the finding that the bottlenecks in the cortisol pathway do cause suppression of cortisol levels may be related to depletion of enzymes involved in cortisol synthesis caused by excessive HPAA activation in MS patients (see review [34]). This hypothesis is supported by data from Kern et al. reporting an increasing cortisol awakening response in MS patients with increasing disability over the course of the disease [76].

#### 3.2.8. 7α-, 7β-, and 16α-Hydroxylating Enzymes (CYP7B1, CYP3A4, CYP3A7)

In general, the C19 Δ^5^ steroids (including their immunoprotective and antidiabetic 7α/β- and 16α-hydroxy-metabolites) mitigate the severity of autoimmune diseases [39,40,41,42,43,44]. However, the autoimmune diseases may suppress the production of adrenal C19 Δ^5^ steroids [39,77]. DHEA controls the Th1/Th2 balance and either favors the Th1 component or attenuates the production of both components [43,78]. The C19 Δ^5^ steroids also suppress cell-mediated immunity and the formation of autoantibodies [41,42,43,44,79], and they may induce restoration of the Th1-dominated cytokine profile. The C19 Δ^5^ steroids and their 7α/β-,16α-hydroxylated metabolites may also counteract the suppression of the primary immune response by glucocorticoids [80].

The first mechanism explaining the immunomodulatory effects of 7α/β-hydroxy-∆^5^-androstanes may be related to the competition of 7-oxygenated androstanes for active sites on 11β-hydroxysteroid dehydrogenase (HSD11B1) catalyzing the conversion of inactive cortisone to immunosuppressive cortisol [81,82].

The second mechanism is underpinned by data showing that the autoimmune response can also be induced by E2, specifically via estrogen receptors. Therefore, the catabolism of androstane steroids such as DHEA and ADIOL, which are estrogen precursors to their 7-oxygenated and 16α-hydroxylated catabolites, can reduce E2 levels. The 7-oxygenated and 16α-hydroxylated catabolites cannot be further converted to bioactive estrogens [83]. Interestingly, E2 stimulates CYP7B1 catalytic activity, mRNA, and the human CYP7B1 reporter gene in human embryonic kidney HEK293 cells and thus may feedback regulate DHEA, E2, and ADIOL levels in human tissues [84]. The ADIOL catabolite 5-androstene-3β,7α,17β-triol, which can be formed either by interconversion from 5-androstene-3β,7α,17β-triol or directly from ADIOL by the catalytic action of CYP3A4 and CYP3A7, is itself immunoprotective despite its low concentrations and high metabolic turnover [85].

Based on the above data, we hypothesize that increased 7α-, 7β-, and 16α-hydroxylation plays a role in the transition from adaptive immunity involving autoimmunity to the innate immune system involving inflammatory processes [8]. Nonetheless, synthetic anti-inflammatory derivatives of 5-androstene-3β,7β,17β-triol suppress the production of inflammatory markers such as C-reactive protein, interleukin 17 (IL-17), TNF-α, and IL-6 signaling as well as the expression of mRNA for IL-6 and matrix metalloproteinase in inflamed tissues, and these steroids also suppress pro-inflammatory cytokines in the lung and intensely stimulate splenic regulatory T cells [86].

The biosynthesis of 7α/β-,16α-hydroxylated Δ^5^ androstanes and their interconversion is illustrated in Figure 7. While the aggregated molar ratios, which may reflect the functioning of the CYP7B1, CYP3A4, and CYP3A7 enzymes, did not show a significant trend related to MS, all four molar ratios related to 7α/β-hydroxylation were significantly lower in patients, although the overall trend was still below the level of statistical significance (*p* = 0.072 by Wilcoxon test), probably due to the low number of events. Therefore, the above data suggests that the impaired 7α/β-hydroxylation of Δ^5^ androstanes forming more potent immunoprotective steroids may be involved in the pathophysiology of MS.

Again, there is the same picture for the formation of immunoprotective 7α/β and 16α-hydroxy-steroids showing a trend to lower activities in patients but their positive correlation with the severity of MS, which may be ascribed to counter-regulatory mechanisms against progression of the disease (Figure 1).

#### 3.2.9. 5α-Reductases (SRD5A1, SRD5A2)

In our present data, 5α-reduced steroids did not show a significant MS-related trend, as well as molar ratios that may reflect the functioning of SRD5As (Figure 4), but transcriptional analyses in the study by Noorbakhsh et al. showed significantly lower SRD5A1 transcripts in the white matter of patients compared to controls [45]. This inconsistency may be due to the different contributions of SRD5A1 and SRD5A2 to the overall conversion of Δ^4^ steroids to 5α-reduced counterparts in the brain and peripheral tissues. Otherwise, however, all indices of MS severity tended to correlate positively with 5α-reduced steroids, suggesting that these findings may also be related to the counter-regulatory effects of 5α-reduced steroids (Figure 1), which include a number of neuroprotective agents [21,45,60,64,67] (see also reviews [46,61,87]). In contrast to the trend toward positive correlations of 5α-reduced steroids with indices of MS severity, the molar ratios that may reflect the functioning of SRD5As were unrelated to MS presence as well as to indices of MS severity. which suggests that changes in SRD5As activities are not associated with MS pathophysiology.

#### 3.2.10. 5β-Reductase (AKR1D1)

Trends in the correlations between 5β-reduced steroids and MS severity indices are not consistent, nor are the relationships between the aforementioned indices and molar ratios, which may reflect the functioning of AKR1D1.

#### 3.2.11. Aldoketoreductase 1C1 (AKR1C1) vs. 17β-Hydroxysteroid Dehydrogenase, Type 2 (HSD17B2)

**Figure 8:**
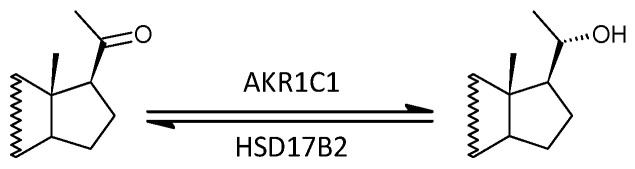
Scheme of the balance between type 1C1 aldoketoreductase (AKR1C1) and type 2 17β-hydroxysteroid dehydrogenase (HSD17B2).

In our present data, the molar ratios that may reflect the functioning of AKR1C1 showed a significant trend related to MS, which was consistent with the results of transcriptional analysis of AKR1C isoforms in the study by Noorbakhsh et al. that also show no significant alteration for the AKR1C1 transcripts in the white matter of patients when compared with controls [45] (Figure 8). Also, the aforementioned molar ratios did not show significant trends in correlations with indices of MS severity.

#### 3.2.12. Aldoketoreductase 1C2 (AKR1C2) vs. 17β-Hydroxysteroid Dehydrogenase, Type 2 and 6 (HSD17B2, HSD17B6)

Of the AKR1C enzymes, AKR1C2 is particularly important for NASs biosynthesis, while HSD3B and SRD5A are upstream of these enzymes in the metabolic pathway to GABAergic steroids. AKR1C2 catalyzes the last step in the reduction of P to pregnanolone isomers, including the most important of them, 3α,5α-THP (allopregnanolone), which is a neuroactive steroid with neuroprotective and neurotrophic properties [45].

Noorbakhsh et al. reported that although levels of the main neurosteroid precursor, pregnenolone, did not differ between clinical groups, a significant reduction in allopregnanolone levels was observed in the white matter of patients compared to controls. Interestingly, these changes were specific for 3α,5α-THP, as the remaining pregnanolone isomers, such as 3α,5β-THP and 3β,5α-THP, were unchanged in patients [45], which was fully consistent with our present data.

In aggregate, the molar ratios, which may reflect a balance between AKR1C2 on one hand and HSD17B2 and HSD17B6 on the other, did not show a significant trend related to MS. However, when comparing the molar ratios of 3α-hydroxy and 3β-hydroxy 5α/β reduced steroids, which may also reflect the balance between AKR1C2 on the one hand and HSD17B2 and HSD17B6 on the other, there was a significant trend towards lower values in patients. These results were also consistent with data from transcriptional analysis in the study by Noorbakhsh et al., which demonstrated significantly lower levels of the AKR1C2 transcript in the brains of MS patients compared to controls. In the same study, western blot analysis of messenger RNA using an antibody against AKR1C2 also showed significantly lower immunoreactivity in patients compared to controls [45].

Otherwise, when comparing the molar ratios of 3α- and 3-oxo- 5α/β-reduced steroids, which may also reflect the balance between AKR1C2 on the one hand and HSD17B2 and HSD17B6 on the other, no significant MS-related trend was found, probably due to the low number of events (Figure 8).

Considering that the enzyme AKR1C2 in the reductive direction and HSD17B2 together with HSD17B6 in the oxidative direction are involved in the interconversion of 3-oxo-5α/β-reduced steroid to their 3α-hydroxy counterparts, and considering the results of transcriptional analyses from the study by Noorbakhsh et al. demonstrating attenuated AKR1C2 function, one can speculate whether the suggested trend towards increased molar ratio of 3α- and 3-oxo-5α/β-reduced steroids in patients (vs. controls) is not due to lower AKR1C2 activity with higher activity of the enzyme HSD17B6, which functions as a 3α/β-epimerase and is (unlike HSD17B2 and AKR1C2) able to convert 3-oxo-5α/β-reduced steroids to 3β-hydroxy 5α/β-reduced steroids.

**Figure 9:**
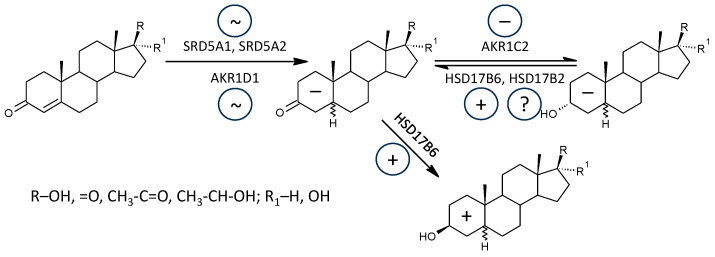
Scheme of the balance between type 1C2 aldoketoreductase (AKR1C2) on one side and type 2 and 6 17β-hydroxysteroid dehydrogenases (HSD17B2 and HSD17B6) on the other.

Perhaps, in addition to the blunted conversion of 3α-hydroxy-5α/β-reduced steroids (caused by impaired functioning of AKR1C2) to intermediates, which are 3-oxo-5α/β-reduced steroids, there is, on the contrary, a rapid conversion of these intermediates to the terminal 3β-hydroxy-5α/β-reduced steroids thanks to the increased activity of HSD17B6 (Figure 9).

The above findings could be relevant in the context of MS pathophysiology, since while 3α-hydroxy-5α/β-reduced steroids are positive modulators of GABA_A_Rs, their 3β-hydroxy counterparts are their antagonists, especially in the sulfated form [88,89,90].

Regarding the associations of the above molar ratios with RS severity indices, we observed trends towards negative correlations that were significant for EDSS, borderline for T25FWT, and absent for HPT9_R and HPT9_L. Thus, it appears that in the case of a balance between AKR1C2 in the reducing direction and HSD17B2 together with HSD17B6 in the oxidative direction, there is no counter-regulation with increasing severity of MS, but on the contrary a further deterioration of the situation from the patient’s perspective.

#### 3.2.13. Aldoketoreductase 1C3 (AKR1C3) vs. 17β-Hydroxysteroid Dehydrogenase, Type 2 (HSD17B2)

AKR1C3 is a reducing enzyme that converts 17-oxo steroids to their 17β-hydroxy counterparts, for example, inactive A to the male sex hormone T, 5α-androstane-3α,17β-diol to the highly potent male hormone 5α-DHT, and inactive estrone to the female sex hormone E2. AKR1C3 is highly expressed in immunocompetent cells, adipose tissue, intestine, smooth muscle, bronchial cells, colon, and liver, but its expression has also been detected in adrenal *zona reticularis* and in a variety of other tissues [91,92,93], http://biogps.org/#goto=genereport&id=8644, accessed on 21 August 2024) (see also reviews [19,94,95]).

CNS inflammation and immune dysfunction are known to play a role in the pathogenesis of MS [1,46,61,96]. Interestingly, besides its involvement in steroidogenesis, AKR1C3 also functions as prostaglandin (PG) F2α synthase. PGF_2α_ and its highly active metabolite 8-iso-PGF_2α_ promote oxidative stress and contribute to the inflammatory environment [97,98,99].

In our present data, the molar ratios that may reflect the balance between reductive AKR1C3 and oxidative HSD17B2 showed a borderline trend towards higher values in patients, which could contribute to the increased incidence of inflammatory responses in MS patients. Moreover, in the context of AKR1C3, it is interesting that despite lower levels of both A and T in female patients compared to controls, the balance between the first and second steroid (catalyzed by AKR1C3 in women) is shifted towards the active androgen T, and even this does not prevent its lower levels in patients (Figure 6).

EDSS and T25FWT but not HPT9_R and HPT9_L tended to negative correlations with the molar ratios that may reflect the balance between reductive AKR1C3 and oxidative HSD17B2, which in this case again shows the contradiction of the relationship to the presence of MS and the relationship to the severity of MS, as in most of the steroidomic alterations described above (except for the relationship to the balance between the activity of the reductive AKR1C2 on the one hand and the activities of the oxidative HSD17B2 and oxidative and epimerizing enzyme HSD17B6 on the other hand, which showed a concordance in the relationship to the presence of MS and the severity of MS).

#### 3.2.14. Potential Clinical Implications of the Findings

The prevailing discordance between the trend in differences between steroidomic alterations in patients compared to controls and the correlations of steroid levels with MS severity may mean that steroid levels could be an efficient indicator of predisposition to MS or incipient MS, but during the progression of MS, differences between steroid levels in patients and controls should diminish as the severity of MS increases, which could worsen the differentiation of patients with more serious MS from controls.

Other possible implications of the study, based on differences in steroidome between MS and controls, as well as correlations of steroidomic data with MS severity, could be the investigation of possibilities of supplementation with certain neuroprotective and immunomodulating steroids, such as progesterone, GABAergic steroids, or 7-hydroxylated Δ^5^ androstanes in patients with MS, or possibilities of treatment with modulators of steroidogenesis enzymes, such as CYP7B1 catalyzing biosynthesis of immunoprotective 7-hydroxylated Δ^5^-androstanes, AKR1C2, which shifts the balance from ineffective or even antagonistic steroids towards neuroprotective GABAergic 3α-hydroxy-5α/β-reduced steroids.

#### 3.2.15. Future Directions

Future research should be focused on mapping the effect of MS treatment in terms of steroidomics, estimating MS predisposition and MS onset based on steroidomic data, and investigating the potential of treatment with neuroprotective/immunoprotective steroids in MS patients. Artificial intelligence could be an effective tool to accomplish these ambitious goals.

#### 3.2.16. Limitations of the Study

Although this study includes a number of steroids and covers most of the steroid metabolic pathways, the study unfortunately does not include 11-deoxycortisol and 11-deoxy-corticosterone, the inclusion of which could lead to the evaluation of changes in CYP21A2 activity, which is the only enzyme involved in the synthesis of cortisol for which data are missing in this study. In the case of estimating the relationship of CYP11B1 activity to MS, although the molar ratios of cortisol/11-deoxycortisol and corticosterone/11-deoxycorticosterone are missing in this study, other suitable markers such as the molar ratios of 11β-hydroxy-androstanes to 11-deoxy-androstanes are available.

The next limitation of this study is the relatively small sample size (25 MS patients, 15 controls), which limits the statistical power and generalizability of the results. We are aware that a larger cohort would strengthen the results. However, despite the limited sample size, our patient population was homogeneous, i.e., only patients of Caucasian origin were included.

Because this study was cross-sectional, it was not possible to determine whether steroid changes are a cause or a consequence of MS. Therefore, further studies on longitudinal data would be valuable. Regarding the proposed changes in the steroidomic pathways, our data were not definitive but were indicative for further studies aimed at validating the proposed pathways.

## 4. Materials and Methods

### 4.1. Subjects

A total of 25 adult female patients aged 39(32, 49) years (shown as median with quartiles) and 15 healthy female age-matched controls aged 38(31, 46) years. The diagnosis of multiple sclerosis was confirmed by cerebrospinal fluid analysis and by magnetic resonance imaging. All of the MS patients fulfilled the revised McDonald criteria from 2017 [100]. The MS patients included in this study had just been diagnosed and had not yet been treated. Patients and controls who had experienced COVID were not included in the present study.

The study was approved by the Ethics Committee of the General University Hospital, Prague, Czech Republic (Approval number: 74/19 Grant AZV VES 2020 VFN, 20 June 2019), and all procedures involving human subjects were conducted following ethical standards set by national and institutional committees on human experimentation and the Helsinki Declaration of 1975, as updated in 2008. The authors guarantee that all research procedures were carried out with the utmost respect for the participant’s safety, well-being, and confidentiality. Participants were examined after signing an informed consent approved by the aforementioned ethics committee. For the evaluation of steroidome, the peripheral blood was withdrawn on fasting in the morning. Blood samples were centrifuged and stored at −20 °C until analyzed.

### 4.2. Steroid Analysis

Steroids and their polar conjugates were measured using our previously described validated GC-MS/MS method [101], with the exception of estradiol, which was quantified using electrochemiluminescence immunoassays (ECLIA), performed on Cobas^®^ Pro, Roche Diagnostics International Ltd. (Rotkreuz, Switzerland).

### 4.3. Statistical Analysis

In the first step, the power transformation parameters were found for each metric variable so that its distribution was as close as possible to the Gaussian distribution. The steroidomic data were evaluated using an ANOVA model as well as multivariate regression with reduced dimensionality known as orthogonal projections to latent structure (OPLS) model. Due to the dependence on age for many of the steroidomic data, the ANOVA model included the factors MS (patients vs. controls) and age (≤38 vs. >38 years of age). Statgraphics Centurion v. XVIII statistical software from Statgraphics Technologies, Inc. (The Plains, VA, USA) was used for power transformations of the original data and for evaluation using the ANOVA model, while SIMCA-P v.12.0 statistical software from Umetrics AB (Umeå, Sweden) was used for OPLS analysis.

The OPLS models were focused on the distinction between controls and patients. Differences between steroid levels in controls and patients found by the ANOVA model (factor MS) and relevant data from the OPLS model are shown in Table 1.

However, from the point of view of diagnosing MS based on steroidomic data, it was more appropriate to use OPLS models that examined the correlation of MS with multiple parameters simultaneously. These models allowed differentiation of patients from controls. The OPLS model, which is multivariate regression with dimensionality reduction, permits the evaluation of relationships between explanatory variables and a number of explanatory variables that may be highly correlated, which is also the case for steroids in metabolic pathways. The presence of the observed pathology is expressed in the OPLS model as the logarithm of the likelihood ratio (the ratio of the probability of the presence of pathology p to the probability of its absence (1-p)), i.e., the logarithm of the likelihood ratio is calculated, which then ranges from -infinity to +infinity. This approach ensures that the prediction of the probability of the presence of pathology is between 0 and 1 (after using a recurrent formula that converts the logarithm of the likelihood ratio to the probability of the presence of pathology).

The variability of the explaining and explained variables is separated into two independent components in the OPLS. The former contains the variability in explaining variables that were shared with the probability of pathology (predictive component), while the orthogonal components express the variability shared in between highly correlated explaining variables (orthogonal components). OPLS identifies significant explanatory variables and their best linear combination to estimate the probability of the presence of pathology. After standardizing the variables, the OPLS model can be expressed as follows:

(1) $$X=T_{p}P_{p}^{T}+T_{o}P_{o}^{T}+E$$

(2) $$Y=T_{p}P_{p}^{T}+F$$

where ***X*** is the matrix with predictors and subjects, ***Y*** is the vector of dependent variables and subjects, ***T****_p_* is the vector of component scores from the single predictive component and subjects extracted from ***Y***; ***T****_o_* is the vector of component scores from the single orthogonal component and subjects extracted from ***X***; ***P****_p_* is the vector of component loadings for the predictive component extracted from ***Y***; ***P****_o_* is the vector of component loadings for the orthogonal component extracted from ***X*** and independent variables, and ***E*** and ***F*** are the error terms.

Significant predictors were selected using the variable importance statistics (VIP). The statistical software SIMCA-P v.12.0 from Umetrics AB (Umeå, Sweden), which was used for OPLS analysis, enabled finding the number of relevant components, the detection of multivariate non-homogeneities, and testing the multivariate normal distribution and homoscedasticity (constant variance).

The algorithm for obtaining the predictions were as follows:

- Transformation of the original data to obtain the values with symmetric distribution and constant variance
- Checking the data homogeneity in predictors using Hotelling’s statistics and the eventual elimination of non-homogeneities
- Testing the relevance of predictors using variable importance statistics and the elimination of irrelevant predictors
- Calculating component loadings for individual variables to evaluate their correlations with the predictive component
- Calculating regression coefficients for the multiple regression model to evaluate the mutual independence of predictors after comparison with the corresponding component loadings from the OPLS model
- Calculating predicted values of the logarithm of the ratio of the probability of pathology presence to the probability of pathology absence (LLR)
- Calculating the probability of the pathology’s presence for individual subjects
- Calculating the sensitivity and specificity of the prediction

The ratio between significantly positive, missing, and significantly negative correlations with MS was evaluated using the one-sample Wilcoxon test with correction for continuity.

## 5. Conclusions

In conclusion, the main outcomes of the present study are

(1) A comprehensive steroidomic analysis was performed in female MS patients compared to female age-matched controls. The MS patients included in this study were newly diagnosed (met the 2017 revised McDonald criteria) and had not yet been treated.
(2) Associations between steroidomic data and indices of MS severity were evaluated.
(3) Most steroids have been studied for the first time in terms of MS.
(4) The results focused on differences between steroidomic data in MS patients and untreated controls, and the results focused on relationships between steroidomic data and MS severity, which were mostly discordant with a tendency to converge to the situation in controls with increasing severity of MS, which was interpreted (in the light of data from the literature) as an intensification of counter-regulatory mechanisms preventing the development of MS with increasing severity of the disease.
(5) A significant trend towards higher ratios of conjugated steroids to their unconjugated counterparts was found in patients, indicating increased SULT2A1 sulfotransferase functioning, which is of particular interest in terms of the balance between excitatory and inhibitory steroid modulators of ionotropic receptors.
(6) An altered metabolic pathway to cortisol was found in patients with decreased conversion of pregnenolone to 17-hydroxypregnenolone and 17-hydroxypregnenolone to 17-hydroxyprogesterone and increased conversion of 17-hydroxypregnenolone to DHEA, resulting in lower levels of 17-hydroxyprogesterone, as well as indications of impaired conversion of 11-deoxy-steroids to 11β-hydroxy-steroids and, finally, reduced conversion of the active glucocorticoid cortisol to its inactive metabolite cortisone.
(7) Despite these metabolic barriers, both cortisol and cortisone levels were higher in patients, and therefore alterations in molar ratios in the cortisol pathway could be explained by depletion of enzymes involved in cortisol synthesis due to overactivation of HPAA, which has been described in MS patients.
(8) Patients showed altered metabolic pathways to both the active androgen testosterone and the active estrogen estradiol, with decreased conversion of DHEA to androstenedione and androstenedione to testosterone, increased conversion of androstenedione to testosterone, and decreased conversion of androstenedione (via unmeasured estrone) to estradiol in the major pathway and testosterone to estradiol in the minor pathway of estradiol synthesis.
(9) Reduced conversion of immunoprotective Δ^5^ androstanes to their more potent 7α/β-hydroxy metabolites was found in patients.
(10) Patients showed lower levels of neuroprotective allopregnanolone compared to controls, as well as a higher ratio of antagonistic 3β-hydroxysteroids to their GABAergic neuroprotective 3α-hydroxy counterparts.

## Figures and Tables

**Table 1:** Steroidomic parameters in women with multiple sclerosis (MS) compared to age-matched controls assessed by ANOVA model with factors Status MS (patients vs. controls) and Age (≤38 vs. >38 years of age), OPLS model and by ordinary multiple regression (MR). Significant alterations (significant in OPLS model and/or in ANOIVA model) are in bold.

Variable Type	Group	Subgroup		Parameter	Orthogonal Predictions to Latent Structure (OPLS)			Multiple Regression (MR)			ANOVA Factor MS (Multiple Sclerosis)				
					Component Loading	t-Statistics	*R^a^*	Regression Coefficient	t-Statistics		Unit	MS−	MS+	Effect size (η_p_^2^)	*p*-Value
EXPLAINING VARIABLES	**Steroids**	Δ^5^ Steroids		Age							years	39 (34, 43)	39 (35, 43)	-	0.898
				Preg							nM	1.2 (0.93, 1.4)	1.4 (1.2, 1.6)	0.026	0.349
				PregS							nM	110 (85, 140)	130 (110, 160)	0.022	0.363
			**↓**	**20α-DHPreg**							**nM**	**4.8 (3.9, 5.7)**	**3.4 (3, 3.9)**	**0.119**	**0.046**
				20α-DHPregS							μM	0.83 (0.68, 1)	0.62 (0.54, 0.72)	0.084	0.097
				17-OH-Preg							nM	3.4 (2.6, 4.4)	3.7 (3, 4.5)	0.004	0.72
				17-OH-PregS							nM	7 (5.4, 8.9)	8.6 (7.2, 10)	0.031	0.315
			**↓**	**16α-OH-Preg**							**nM**	**0.58 (0.46, 0.73)**	**0.3 (0.26, 0.36)**	**0.262**	**0.003**
				DHEA							nM	5 (4, 6.2)	5.6 (4.7, 6.6)	0.011	0.539
				DHEAS							μM	2.2 (1.6, 3)	2.6 (2.1, 3.2)	0.009	0.57
				7α-OH-DHEA							nM	1.2 (0.94, 1.4)	0.95 (0.79, 1.1)	0.032	0.309
			**↓**	**7β-OH-DHEA**							**nM**	**0.69 (0.55, 0.87)**	**0.36 (0.29, 0.44)**	**0.233**	**0.005**
				Adiol							nM	1.5 (1.2, 1.9)	1.8 (1.5, 2.1)	0.015	0.461
				AdiolS							μM	1.1 (0.8, 1.5)	0.68 (0.54, 0.86)	0.088	0.084
				3β,7α,17β-AT							nM	0.19 (0.14, 0.25)	0.2 (0.16, 0.24)	<0.001	0.912
				3β,7β,17β-AT							nM	0.19 (0.14, 0.25)	0.18 (0.14, 0.22)	0.001	0.837
				3β,16α,17β-AT							pM	160 (120, 220)	170 (130, 220)	<0.001	0.899
				3β,16α,17β-ATC							nM	78 (59, 100)	94 (75, 120)	0.015	0.471
		Δ^4^ Steroids		P							pM	180 (110, 310)	130 (86, 190)	0.014	0.476
				20α-DHP							nM	0.23 (0.15, 0.33)	0.25 (0.19, 0.34)	0.003	0.722
				20α-DHPC							nM	1.6 (1.2, 2.2)	1.6 (1.2, 2)	<0.001	0.995
			**↓**	**17-OH-P**	**−0.377**	−10.4 **	**−0.772**	**−0.110**	−4.28 **		**nM**	**1.2 (0.87, 1.6)**	**0.67 (0.53, 0.86)**	**0.106**	**0.046**
				17-OH-20α-DHP							nM	0.75 (0.56, 0.99)	0.46 (0.37, 0.58)	0.09	0.072
				17-OH-20α-DHPC							nM	6.5 (4.9, 8.6)	8 (6.4, 9.9)	0.02	0.412
			**↓**	**16α-OH-P**	**−0.351**	−3.88 **	**−0.720**	**−0.092**	−4.26 **		**nM**	**0.46 (0.35, 0.59)**	**0.28 (0.22, 0.34)**	**0.138**	**0.037**
			**↓**	**A**							**nM**	**3 (2.6, 3.4)**	**2 (1.8, 2.3)**	**0.215**	**0.005**
			**↓**	**T**							**nM**	**0.59 (0.48, 0.72)**	**0.38 (0.31, 0.46)**	**0.135**	**0.032**
			**↓**	**E2**							**pM**	**260 (160, 430)**	**100 (70, 150)**	**0.123**	**0.033**
EXPLAINING VARIABLES	**Steroids**	20-oxo-5α/β-Reduced pregnanes		5α-DHP							pM	98 (62, 150)	89 (61, 130)	0.002	0.809
			**↓**	**3α,5α-THP**							**pM**	**200 (160, 260)**	**120 (100, 150)**	**0.138**	**0.04**
				3α,5α-THPC							nM	7.2 (5.5, 9.6)	5.8 (4.7, 7.1)	0.024	0.346
				3β,5α-THP							pM	120 (97, 160)	100 (86, 120)	0.024	0.388
				3β,5α-THPC							nM	11 (9.6, 13)	11 (10, 13)	<0.001	0.997
				3α,5β-THP							pM	73 (49, 110)	67 (49, 92)	0.002	0.813
				3α,5β-THPC							nM	19 (16, 23)	15 (13, 17)	0.061	0.153
				3α,5β-THPC							nM	2.5 (2.1, 3)	3.2 (2.8, 3.7)	0.075	0.117
				(3α,5α,17-PDC							nM	2.1 (1.7, 2.6)	2 (1.7, 2.4)	0.001	0.846
			**↓**	**3α,5β,17-PD**	**−0.435**	−10.46 **	**−0.771**	**−0.130**	−3.39 **		**pM**	**56 (37, 85)**	**24 (16, 36)**	**0.137**	**0.04**
			**↓**	**3α,5β,17-PDC**							**nM**	**14 (12, 15)**	**8.4 (7.4, 9.5)**	**0.328**	**<0.001**
				5α,20α-THP							pM	150 (110, 200)	140 (110, 180)	0.001	0.839
				5α,20α-THPC							nM	0.39 (0.29, 0.52)	0.29 (0.24, 0.37)	0.032	0.28
		20α-Hydroxy-5α/β-reduced pregnanes		3α,5α,20α-PD							nM	0.3 (0.22, 0.4)	0.34 (0.27, 0.43)	0.006	0.644
				3α,5α,20α-PDC							nM	25 (18, 34)	28 (22, 35)	0.007	0.625
				3β,5α,20α-PD							nM	3.4 (2.5, 4.5)	2.6 (2, 3.3)	0.032	0.353
				3β,5α,20α-PDC							nM	530 (420, 680)	390 (330, 460)	0.072	0.126
				5β,20α-THPC							nM	0.64 (0.5, 0.86)	0.5 (0.42, 0.61)	0.033	0.281
				3α,5β,20α-PD							pM	110 (82, 150)	90 (69, 120)	0.019	0.427
				3α,5β,20α-PDC							nM	17 (13, 21)	16 (14, 19)	<0.001	0.912
				3β,5β,20α-PD							nM	0.16 (0.12, 0.22)	0.16 (0.13, 0.2)	<0.001	0.904
				3β,5β,20α-PDC							nM	10 (8.3, 13)	15 (13, 19)	0.105	0.066
			**↓**	**3α,5α,17,20α-PT**	**−0.389**	−10.24 **	**−0.796**	**−0.083**	−7.01 **		**pM**	**170 (110, 250)**	**100 (71, 140)**	**0.056**	**0.152**
				3α,5α,17,20α-PTC							nM	41 (29, 56)	42 (32, 53)	<0.001	0.922
				3β,5α,17,20α-PT							pM	120 (76, 190)	140 (99, 200)	0.006	0.653
			**↑**	**3β,5α,17,20α-PTC**							**nM**	**3.1 (2.2, 4.3)**	**6 (4.4, 8.4)**	**0.114**	**0.044**
			**↓**	**3α,5β,17,20α-PT**	**−0.411**	−7.43 **	**−0.841**	**−0.091**	−3.95 **		**nM**	**1.7 (1.3, 2.2)**	**1.3 (0.98, 1.6)**	**0.036**	**0.254**
				3α,5β,17,20α-PTC							nM	100 (80, 130)	89 (72, 110)	0.013	0.496
EXPLAINING VARIABLES	**Steroids**	5α/β-Reduced androstanes	**↓**	**5α-DHA**	**−0.384**	−7.2 **	**−0.786**	**−0.101**	−3.78 **		**nM**	**0.23 (0.2, 0.27)**	**0.17 (0.15, 0.19)**	**0.146**	**0.024**
			**↑**	**3α,5α-THA**							**nM**	**0.32 (0.28, 0.38)**	**0.49 (0.44, 0.55)**	**0.256**	**0.003**
				3α,5α-THAC							μM	0.77 (0.58, 1)	1 (0.79, 1.2)	0.034	0.299
				3β,5α-THA							nM	0.22 (0.17, 0.27)	0.21 (0.17, 0.25)	0.002	0.808
				3β,5α-THAC							nM	300 (210, 410)	320 (250, 410)	0.002	0.806
				3α,5β-THA							nM	0.15 (0.12, 0.18)	0.15 (0.13, 0.18)	<0.001	0.956
				3α,5β-THAC							nM	59 (44, 77)	71 (57, 86)	0.015	0.466
				3β,5β-THAC							nM	17 (12, 24)	26 (20, 33)	0.048	0.178
				5α-DHT							nM	0.16 (0.13, 0.19)	0.16 (0.14, 0.19)	<0.001	0.889
				5α-DHTC							nM	1.4 (1.1, 1.6)	1.5 (1.3, 1.7)	0.006	0.65
				3α,5α,17β-AD							pM	66 (52, 84)	56 (47, 68)	0.016	0.452
				3α,5α,17β-ADC							nM	29 (22, 38)	28 (23, 35)	<0.001	0.956
				3β,5α,17β-AD							pM	20 (16, 25)	21 (18, 25)	0.003	0.764
				3β,5α,17β-ADC							nM	64 (47, 84)	67 (53, 82)	<0.001	0.864
				3α,5β,17β-ADC							nM	6.8 (5.3, 8.6)	6.4 (5.2, 7.8)	0.002	0.805
			**↑**	**3β,5β,17β-ADC**							**nM**	**0.4 (0.3, 0.51)**	**0.65 (0.53, 0.78)**	**0.139**	**0.033**
		Corticoids + 11β-OH-androst.	**↑**	**F**	**0.162**	**0.99**	**0.330**	**0.087**	**1.6**		**nM**	**300 (250, 350)**	**440 (380, 500)**	**0.173**	**0.009**
			**↑**	**E**							**nM**	**110 (100, 120)**	**140 (120, 150)**	**0.144**	**0.032**
				B							nM	12 (8.8, 17)	12 (9.7, 16)	<0.001	0.974
				11β-OH-A							nM	42 (36, 48)	39 (35, 43)	0.014	0.513
			**↓**	**11β-OH-3α,5α-THA**	**−0.304**	−5.24 **	**−0.625**	**−0.073**	−3.45 **		**nM**	**1.9 (1.5, 2.4)**	**1.1 (0.91, 1.4)**	**0.129**	**0.029**
				11β-OH-3α,5α-THAC							nM	33 (27, 40)	27 (22, 31)	0.045	0.223
				11β-OH-3β,5α-THA							pM	95 (67, 130)	54 (41, 71)	0.085	0.081
				11β-OH-3β,5α-THAC							nM	0.96 (0.76, 1.2)	1.3 (1.1, 1.6)	0.063	0.142
				11β-OH-3α,5β-THA							nM	1.7 (1.5, 2)	1.3 (1.1, 1.5)	0.073	0.105
				11β-OH-3α,5β-THAC							nM	13 (10, 17)	8.5 (6.7, 11)	0.088	0.075
EXPLAINED VARIABLE				MS patient vs. control (LLR*^b^*)	1.000	3.66 **	0.560								
				Explained variability = 31.4% (28.2% after cross-validation), Sensitivity = 0.875(0.743–1), Specificity = 0.75(0.505–0.995)											
EXPLAINING VARIABLES	**CYP17A1**	hydroxylase + lyase		DHEA/Preg								4.8 (4.1, 5.7)	5.3 (4.7, 6)	0.016	0.48
				DHEA/PregC								18 (16, 21)	20 (18, 23)	0.02	0.394
			**↑**	**DHEA/20α-DHPreg**	**0.272**	3.49 **	**0.583**	**0.063**	2.8 *			**1.7 (1.5, 2)**	**2 (1.8, 2.2)**	**0.025**	**0.36**
				DHEA/20α-DHPregC								2.8 (2.3, 3.4)	3.8 (3.3, 4.4)	0.073	0.097
				A/P								13 (8.1, 21)	18 (13, 25)	0.017	0.432
				A/20α-DHP								8.9 (6.1, 13)	8.5 (6.3, 11)	<0.001	0.871
				5α-DHA/5α-DHP								2.3 (1.6, 3.2)	2.9 (2.2, 4)	0.022	0.444
			**↑**	**5α-DHA/5α,20α-THP**								**1.3 (1.1, 1.6)**	**1.9 (1.7, 2.2)**	**0.137**	**0.026**
			**↑**	**3α,5α-THA/3α,5α-THP**								**2 (1.6, 2.5)**	**3.1 (2.6, 3.8)**	**0.116**	**0.046**
			**↓**	**3α,5α-THA/3α,5α,20α-PD**	**−0.091**	**−1.14**	**−0.196**	**−0.049**	−2.7 *			**1.6 (1.2, 2.1)**	**1.2 (0.95, 1.5)**	**0.035**	**0.256**
			**↑**	**3α,5α-THA/3α,5α-THP, C**	**0.357**	9.24 **	**0.765**	**0.089**	2.87 *			**89 (69, 110)**	**180 (150, 220)**	**0.241**	**0.002**
			**↑**	**3α,5α-THA/3α,5α,20α-PD, C**	**0.394**	14.89 **	**0.846**	**0.081**	3.12 **			**20 (15, 26)**	**36 (30, 43)**	**0.173**	**0.013**
				3β,5α-THA/3β,5α-THP								2.2 (1.7, 2.8)	2.1 (1.7, 2.5)	0.002	0.81
				3β,5β-THA/3α,5α,20α-PD							10^3^	94 (77, 120)	69 (58, 82)	0.107	0.104
			**↑**	**3β,5α-THA/3β,5α-THP, C**	**0.393**	11.86 **	**0.843**	**0.098**	5.56 **			**27 (23, 32)**	**34 (30, 38)**	**0.076**	**0.12**
			**↑**	**3β,5β-THA/3α,5α,20α-PD, C**	**0.400**	6.59 **	**0.859**	**0.086**	4.48 **			**0.73 (0.56, 0.93)**	**0.82 (0.67, 0.99)**	**0.008**	**0.602**
				3α,5β-THA/3α,5β-THP								2.7 (1.9, 3.7)	2.8 (2.1, 3.6)	<0.001	0.89
				3α,5β-THA/3α,5β,20α-PD								1.3 (1.1, 1.6)	1.9 (1.6, 2.2)	0.096	0.074
			**↑**	**3α,5β-THA/3α,5β-THP, C**	**0.393**	11.86 **	**0.843**	**0.098**	5.56 **			**2.6 (2, 3.3)**	**4.3 (3.6, 5.1)**	**0.136**	**0.021**
			**↑**	**3α,5β-THA/3α,5β,20α-PD, C**	**0.400**	6.59 **	**0.859**	**0.086**	4.48 **			**2.6 (1.9, 3.4)**	**3.9 (3.2, 4.7)**	**0.079**	**0.087**
			**↑**	**3β,5β-THA/3β,5β-THP, C**	**0.425**	9.6 **	**0.911**	**0.136**	3.78 **			**0.75 (0.62, 0.91)**	**1.6 (1.4, 1.9)**	**0.387**	**<0.001**
			**↑**	**3β,5β-THA/3β,5β,20α-PD, C**	**0.416**	11.86 **	**0.892**	**0.118**	5.01 **			**0.73 (0.52, 1)**	**1.5 (1.2, 1.9)**	**0.154**	**0.014**
												3.8 (3, 4.8)	4.1 (3.5, 4.9)	0.005	0.669
EXPLAINED VARIABLE				MS patient vs. control (LLR)	1.000	4.79 **	0.570								
				Explained variability = 32.5% (29% after cross-validation), Sensitivity = 0.84(0.696–0.984), Specificity = 0.833(0.622–1)											
EXPLAINING VARIABLES	**CYP17A1**	hydroxylase	**↓**	**17-OH-Preg/Preg**								**4.3 (3.4, 5.4)**	**2.9 (2.5, 3.3)**	**0.124**	**0.041**
				17-OH-Preg/Preg, C							10^3^	74 (63, 86)	72 (63, 80)	0.002	0.793
				17-OH-P/P								5.4 (3.6, 7.6)	5 (3.7, 6.6)	0.001	0.842
			**↓**	**17-OH-20α-DHP/20α-DHP**	**−0.599**	−10.72 **	**−0.800**	**−0.174**	−2.67 *			**2.6 (1.9, 3.4)**	**2 (1.6, 2.5)**	**0.025**	**0.346**
				17-OH-20α-DHP/20α-DHP, C								4.6 (3.8, 5.6)	5.5 (4.8, 6.3)	0.032	0.309
				3α,5α,17-PD/3α,5α-THP, C								0.25 (0.2, 0.3)	0.3 (0.26, 0.35)	0.045	0.237
			**↓**	**3α,5β,17-PD/3α,5β-THP**	**−0.630**	−9.57 **	**−0.842**	**−0.219**	−2.6 *			**0.93 (0.61, 1.5)**	**0.4 (0.27, 0.6)**	**0.145**	**0.046**
				3α,5β,17-PD/3α,5β-THP, C								0.68 (0.58, 0.81)	0.52 (0.47, 0.58)	0.109	0.061
			**↓**	**3α,5α,17,20α-PT/3α,5α,20α-PD**	**−0.499**	−5.21 **	**−0.677**	**−0.160**	−6.61 **			**0.6 (0.4, 0.88)**	**0.31 (0.21, 0.43)**	**0.094**	**0.073**
				3α,5α,17,20α-PT/3α,5α,20α-PD, C								0.95 (0.64, 1.4)	1.4 (1, 1.8)	0.032	0.286
				3β,5α,17,20α-PT/3β,5α,20α-PD							10^3^	61 (40, 91)	64 (45, 91)	<0.001	0.876
			**↑**	**3β,5α,17,20α-PT/3β,5α,20α-PD, C**							**10^3^**	**7.1 (4.8, 10)**	**15 (11, 22)**	**0.127**	**0.039**
				3α,5β,17,20α-PT/3α,5β,20α-PD								14 (11, 19)	12 (9.5, 14)	0.022	0.37
				3α,5β,17,20α-PT/3α,5β,20α-PD, C								5 (3.9, 6.3)	5 (4.1, 5.9)	<0.001	0.98
				F/B								28 (21, 35)	36 (30, 43)	0.039	0.234
EXPLAINED VARIABLE				MS patient vs. control (LLR)	1.000	2.34 *	0.434								
				Explained variability = 18.8% (14.5% after cross-validation), Sensitivity = 0.7(0.536–0.864), Specificity = 0.556(0.231–0.88)											
EXPLAINING VARIABLES	**CYP17A1**	lyase	**↑**	**DHEA/17-OH-Preg**	**0.375**	7 **	**0.662**	**0.122**	2.78 *			**1.2 (1.1, 1.3)**	**1.6 (1.4, 1.7)**	**0.176**	**0.011**
				DHEA/17-OH-Preg, C								250 (210, 300)	270 (230, 310)	0.008	0.615
			**↑**	**A/17-OH-P**	**0.451**	8.86 **	**0.796**	**0.121**	3.81 **			**1.4 (1.1, 1.8)**	**2.6 (2.1, 3.2)**	**0.187**	**0.011**
				A/17-OH-20α-DHP								3.6 (3, 4.5)	4.2 (3.6, 5)	0.016	0.442
				3α,5α-THA/3α,5α,17-PD, C								430 (320, 590)	450 (350, 580)	<0.001	0.871
			**↓**	**3α,5β-THA/3α,5β,17-PD**	**−0.429**	−5.65 **	**−0.684**	**−0.130**	−4.12 **		**10^3^**	**320 (220, 470)**	**140 (98, 200)**	**0.148**	**0.032**
			**↓**	**3α,5β-THA/3α,5β,17-PD, C**	**−0.424**	−9.39 **	**−0.748**	**−0.113**	−3.17 **			**0.24 (0.18, 0.31)**	**0.13 (0.11, 0.16)**	**0.159**	**0.015**
			**↓**	**3α,5α-THA/3α,5α,17,20α-PT**								**0.46 (0.34, 0.6)**	**0.24 (0.19, 0.31)**	**0.148**	**0.022**
				3α,5α-THA/3α,5α,17,20α-PT, C							10^3^	51 (35, 74)	41 (31, 56)	0.01	0.545
				3β,5α-THA/3β,5α,17,20α-PT								0.54 (0.36, 0.79)	0.79 (0.58, 1.1)	0.035	0.271
			**↑**	**3β,5α-THA/3β,5α,17,20α-PT, C**							10^3^	**12 (8.8, 16)**	**23 (18, 31)**	**0.165**	**0.019**
			**↓**	**3α,5β-THA/3α,5β,17,20α-PT**	**−0.383**	−8.59 **	**−0.679**	**−0.132**	−3.47 **			**11 (9.3, 13)**	**6 (5.2, 6.8)**	**0.342**	**<0.001**
			**↓**	**3α,5β-THA/3α,5β,17,20α-PT, C**	**−0.397**	−7.95 **	**−0.699**	**−0.102**	**−1.87**			**1.9 (1.5, 2.4)**	**1.1 (0.96, 1.3)**	**0.165**	**0.016**
			**↓**	**11β-OH-A/F**							**10^3^**	**130 (120, 150)**	**110 (98, 120)**	**0.131**	**0.039**
EXPLAINED VARIABLE				MS patient vs. control (LLR)	1.000	5.28 **	0.521								
				Explained variability = 27.1% (21.3% after cross-validation), Sensitivity = 0.75(0.59–0.91), Specificity = 0.6(0.296–0.904)											
EXPLAINING VARIABLES	**HSD3B1, HSB3B2**			P/Preg							10^3^	140 (89, 230)	93 (69, 130)	0.028	0.312
			**↓**	**P/PregC**	**−0.351**	−7.09 **	**−0.614**	**−0.110**	−2.88 *		**10^3^**	**1.6 (0.94, 2.8)**	**0.88 (0.59, 1.3)**	**0.043**	**0.211**
				20α-DHP/20α-DHPreg							10^3^	53 (42, 68)	67 (56, 83)	0.032	0.273
				20α-DHP/20α-DHPreg, C							10^3^	0.3 (0.27, 0.32)	0.3 (0.29, 0.32)	0.006	0.649
			**↓**	**17-OH-P/17-OH-Preg**	**−0.459**	−7.62 **	**−0.803**	**−0.158**	−3.14 **			**0.24 (0.19, 0.31)**	**0.18 (0.15, 0.21)**	**0.061**	**0.153**
			**↓**	**17-OH-P/17-OH-Preg, C**	**−0.472**	−7.59 **	**−0.825**	**−0.205**	−5.68 **		**10^3^**	**240 (170, 340)**	**72 (57, 90)**	**0.378**	**<0.001**
				16α-OH-P/16α-OH-Preg								0.86 (0.69, 1.1)	0.73 (0.62, 0.88)	0.018	0.431
			**↓**	**A/DHEA**	**−0.437**	−4.66 **	**−0.765**	**−0.180**	−7.24 **		**10^3^**	**440 (390, 520)**	**360 (330, 390)**	**0.101**	**0.059**
			**↓**	**A/DHEAC**	**−0.402**	−9.89 **	**−0.704**	**−0.117**	−7.32 **		**10^3^**	**1.2 (0.9, 1.5)**	**0.81 (0.67, 0.97)**	**0.079**	**0.108**
			**↓**	**T/Adiol**	**−0.338**	−5.7 **	**−0.591**	**−0.150**	−3.41 **		**10^3^**	**320 (260, 390)**	**270 (230, 310)**	**0.032**	**0.294**
				T/AdiolC							10^3^	0.41 (0.31, 0.53)	0.54 (0.44, 0.67)	0.044	0.244
EXPLAINED VARIABLE				**MS patient vs. control (LLR)**	**1.000**	4.5 **	**0.673**								
				Explained variability = 45.2% (41.2% after cross-validation), Sensitivity = 0.846(0.707–0.985), Specificity = 0.889(0.684–1)											
EXPLAINING VARIABLES	**CYP** **19A1**		**↓**	**E2/A**	**−0.579**	−73.7 **	**−0.987**	**−0.152**	−4.35 **		**10^3^**	**99 (64, 160)**	**40 (29, 55)**	**0.149**	**0.02**
			**↓**	**E2/T**	**−0.569**	−41.6 **	**−0.969**	**−0.153**	−6.07 **		**10^3^**	**500 (300, 860)**	**200 (140, 290)**	**0.107**	**0.048**
			**↓**	**E2/(A+T)**	**−0.584**	**−148**	**−0.996**	**−0.156**	−4.89 **		**10^3^**	**82.1 (52.6, 133)**	**31.9 (23.1, 44.2)**	**0.154**	**0.02**
EXPLAINED VARIABLE				**MS patient vs. control (LLR)**	**1.000**	3.57 **	**0.454**								
				Explained variability = 20.6% (18.8% after cross-validation), Sensitivity = 0.708(0.526–0.89), Specificity = 0.583(0.304–0.862)											
EXPLAINING VARIABLES	**Conjugated/unconjugated steroids (C/U)**	Δ^5^ + Δ^4^ Steroids		Preg								99 (78, 130)	100 (84, 120)	<0.001	0.955
				20α-DHPreg								140 (120, 170)	190 (170, 230)	0.105	0.05
				17-OH-Preg								1.6 (1.2, 2.1)	2.5 (2.1, 3)	0.104	0.052
				DHEA	0.196	1.21	0.319	0.117	1.84			430 (340, 540)	370 (310, 450)	0.015	0.481
				Adiol								570 (420, 780)	430 (350, 540)	0.027	0.314
				3β,16α,17β-AT								590 (440, 790)	440 (360, 550)	0.038	0.255
				20α-DHP								6.8 (4.2, 11)	7 (4.8, 10)	<0.001	0.954
				17-OH-20α-DHP								12 (7.8, 17)	17 (13, 24)	0.033	0.272
		5α/β-Reduced pregnanes		3α,5α-THP								42 (31, 58)	35 (28, 45)	0.013	0.501
				3β,5α-THP								96 (72, 130)	110 (86, 130)	0.005	0.677
				3α,5β-THP								340 (240, 460)	250 (180, 330)	0.033	0.317
			**↑**	**3α,5β,17-PD**	**0.452**	5.83 **	**0.648**	**0.151**	3.17 **			**210 (150, 300)**	**360 (260, 520)**	**0.082**	**0.112**
				5α,20α-THP								2.3 (1.7, 3)	2.2 (1.8, 2.7)	<0.001	0.926
			**↓**	**3α,5α,20α-PD**	**−0.334**	−2.5 *	**−0.544**	**−0.111**	**−1.72**			**130 (81, 210)**	**79 (58, 110)**	**0.039**	**0.239**
				3β,5α,20α-PD								160 (120, 220)	230 (180, 300)	0.05	0.237
				3α,5β,20α-PD								180 (130, 260)	160 (130, 210)	0.004	0.694
			**↑**	**3β,5β,20α-PD**								**63 (45, 91)**	**140 (100, 190)**	**0.159**	**0.022**
			**↑**	**3α,5α,17,20α-PT**	**0.274**	2.38 *	**0.448**	**0.112**	1.91 *			**160 (110, 230)**	**310 (240, 410)**	**0.12**	**0.045**
			**↑**	**3β,5α,17,20α-PT**								**22 (18, 26)**	**41 (36, 47)**	**0.331**	**<0.001**
			**↑**	**3α,5β,17,20α-PT**	**0.403**	3.49 **	**0.656**	**0.138**	4.49 **			**60 (49, 73)**	**81 (69, 95)**	**0.075**	**0.096**
		5α/β-Reduced androstanes		3α,5α-THA							10^−3^	1.6 (1.3, 2.1)	1.9 (1.5, 2.3)	0.014	0.496
				3β,5α-THA							10^−3^	1.2 (1, 1.4)	1.4 (1.2, 1.6)	0.024	0.381
				3α,5β-THA								310 (260, 370)	420 (370, 490)	0.111	0.058
				5α-DHT								9.7 (7.3, 13)	7.3 (5.9, 9.1)	0.037	0.261
				3α,5α,17β-AD							10^−3^	0.48 (0.38, 0.61)	0.45 (0.37, 0.55)	0.004	0.711
				3β,5α,17β-AD							10^−3^	3.3 (2.5, 4.5)	2.6 (2.1, 3.3)	0.027	0.363
			**↑**	**11β-OH-3α,5α-THA**	**0.457**	6.1 **	**0.746**	**0.173**	4.61 **			**9 (7, 11)**	**18 (15, 22)**	**0.237**	**0.003**
			**↑**	**11β-OH-3β,5α-THA**	**0.469**	7.08 **	**0.767**	**0.195**	2.99 *			**14 (12, 17)**	**23 (21, 26)**	**0.301**	**<0.001**
				11β-OH-3α,5β-THA								6.4 (4.9, 8.3)	7.1 (5.9, 8.6)	0.007	0.632
EXPLAINED VARIABLE				MS patient vs. control (LLR)	1.000	4.33 **	0.630								
				Explained variability = 39.7% (31.7% after cross-validation), Sensitivity = 0.875(0.743–1), Specificity = 0.714(0.478–0.951)											
EXPLAINING VARIABLES	**CYP11B1**			11β-OH-A/A								17 (14, 20)	19 (16, 23)	0.018	0.43
			**↓**	**11β-OH-3α,5α-THA/3α,5α-THA**	**−0.693**	−11.66 **	**−0.883**	**−0.245**	−4.67 **			**5.2 (4.1, 6.4)**	**3.3 (2.7, 4)**	**0.119**	**0.034**
				11β-OH-3α,5α-THA/3α,5α-THA, C							10^3^	45 (35, 59)	35 (28, 43)	0.038	0.265
				11β-OH-3β,5α-THA/3β,5α-THA							10^3^	350 (270, 440)	220 (180, 270)	0.107	0.055
				11β-OH-3β,5α-THA/3β,5α-THA, C							10^3^	3.5 (2.7, 4.6)	3.7 (3, 4.7)	0.002	0.805
			**↓**	**11β-OH-3α,5β-THA/3α,5β-THA**	**−0.722**	−11.82 **	**−0.905**	**−0.274**	−3.99 **			**10 (8.6, 12)**	**7.5 (6.5, 8.7)**	**0.102**	**0.057**
			**↓**	**11β-OH-3α,5β-THA/3α,5β-THA, C**							**10^3^**	**200 (150, 270)**	**120 (94, 140)**	**0.121**	**0.035**
EXPLAINED VARIABLE				MS patient vs. control (LLR)	1.000	2.05 *	0.470								
				Explained variability = 22% (19.6% after cross-validation), Sensitivity = 0.708(0.526–0.89), Specificity = 0.583(0.304–0.862)											
EXPLAINING VARIABLES	**CYP7B1, CYP3A4, CYP3A7**		**↓**	**7α-OH-DHEA/DHEA**	**−0.533**	−22.64 **	**−0.931**	**−0.130**	−3.78 **		**10^3^**	**170 (140, 200)**	**140 (120, 160)**	**0.044**	**0.211**
			**↓**	**3β,7α,17β-AT/Adiol**	**−0.497**	−12.96 **	**−0.869**	**−0.126**	−5.34 **		**10^3^**	**220 (180, 260)**	**170 (150, 190)**	**0.076**	**0.116**
			**↓**	**7β-OH-DHEA/DHEA**	**−0.500**	−9.76 **	**−0.874**	**−0.149**	−4.77 **		**10^3^**	**94 (76, 120)**	**53 (44, 62)**	**0.226**	**0.005**
			**↓**	**3β,7β,17β-AT/Adiol**	**−0.474**	−7.12 **	**−0.829**	**−0.124**	−3.81 **		**10^3^**	**120 (110, 140)**	**110 (94, 120)**	**0.049**	**0.2**
			**↓**	**16α-OH-Preg/Preg**							**10^3^**	**400 (310, 510)**	**210 (180, 240)**	**0.248**	**0.003**
				3β,16α,17β-AT/Adiol							10^3^	99 (80, 130)	77 (66, 92)	0.055	0.205
			**↑**	**3β,16α,17β-AT/Adiol, C**							**10^3^**	**60 (49, 75)**	**98 (82, 120)**	**0.164**	**0.017**
				16α-OH-P/P								1.8 (1.1, 2.7)	1.7 (1.2, 2.3)	0.001	0.845
EXPLAINED VARIABLE				MS patient vs. control (LLR)	1.000	3.8 **	0.463								
				Explained variability = 21.4% (19.1% after cross-validation), Sensitivity = 0.68(0.497–0.863), Specificity = 0.455(0.16–0.749)											
EXPLAINING VARIABLES	**HSD11B1**			7β-OH-DHEA/7α-OH-DHEA								0.5 (0.43, 0.56)	0.46 (0.41, 0.51)	0.014	0.487
			**↑**	**3β,7β,17β-AT/3β,7α,17β-AT/Adiol**	**0.323**	**0.95**	**0.314**	**0.250**	2.08 *			**0.59 (0.53, 0.65)**	**0.73 (0.69, 0.78)**	**0.191**	**0.011**
			**↑**	**F/E**	**0.948**	7.82 **	**0.921**	**0.609**	6.96 **			**2.6 (2.3, 2.9)**	**3.6 (3.3, 3.9)**	**0.239**	**0.003**
EXPLAINED VARIABLE				MS patient vs. control (LLR)	0.948	7.82 **	0.921								
				Explained variability = 40.8% (38.2% after cross-validation), Sensitivity = 0.8(0.643–0.957), Specificity = 0.727(0.464–0.99)											
EXPLAINING VARIABLES	**SRD5A1, SRD5A2**			(5α-DHP+3α/β,5α-THP)/P								2.9 (1.7, 4.8)	2.3 (1.5, 3.4)	0.011	0.583
				3α/β,5α-THP, C/P								100 (60, 170)	130 (89, 200)	0.008	0.587
				(5α,20α-THP+3α/β,5α,20α-PD)/20α-DHP								15 (11, 20)	9.8 (7.6, 13)	0.099	0.11
				(5α,20α-THP+3α/β,5α,20α-PD)/20α-DHP, C								350 (250, 500)	280 (220, 370)	0.015	0.454
				3α,5α,17-PDC/17-OH-P								1.8 (1.4, 2.3)	2.3 (1.9, 2.8)	0.033	0.279
				3α,5α,17,20α-PT/17-OH-20α-DHP								0.47 (0.33, 0.66)	0.5 (0.38, 0.65)	0.001	0.824
				3α,5α,17,20α-PT/17-OH-20α-DHP, C								7.3 (4.8, 11)	6.5 (4.6, 8.8)	0.003	0.73
				(5α-DHA+3α/β,5α-THA)/A								0.37 (0.32, 0.43)	0.42 (0.38, 0.46)	0.024	0.372
			**↑**	**3α/β,5α-THA, C/A**								**400 (310, 520)**	**700 (560, 870)**	**0.151**	**0.025**
				(5α-DHT+3α/β,5α-AD)/T								0.54 (0.43, 0.67)	0.58 (0.5, 0.68)	0.005	0.674
				(5α-DHT+3α/β,5α-AD), C/T								3 (2.2, 3.8)	4 (3.3, 4.8)	0.047	0.197
			**↓**	**11β-OH-3α/β,5α-THA/A**							**10^3^**	**45 (36, 57)**	**27 (22, 33)**	**0.151**	**0.023**
				11β-OH-3α/β,5α-THA, C/A								0.81 (0.62, 1)	0.81 (0.66, 0.98)	<0.001	0.983
EXPLAINED VARIABLE				MS patient vs. control (LLR)											
				Not relevant											
EXPLAINING VARIABLES	**AKR1D1**			3α,5β-THP/P								0.35 (0.21, 0.57)	0.42 (0.28, 0.63)	0.006	0.67
				3α/β,5β-THP, C/P								140 (83, 220)	140 (98, 210)	<0.001	0.905
				3α/β,5β,20α-PD/20α-DHP								1.3 (0.93, 1.8)	1.4 (1.1, 1.8)	0.002	0.784
				(5β,20α-THP+3α/β,5β,20α-PD)/20α-DHP, C								30 (21, 42)	22 (17, 29)	0.026	0.325
				3α,5β,17-PD/17-OH-P							10^3^	51 (35, 74)	38 (27, 53)	0.022	0.414
				3α,5β,17-PDC/17-OH-P								9.1 (7.8, 11)	12 (11, 14)	0.108	0.054
				3α,5β,17,20α-PT/17-OH-20α-DHP								2.2 (1.9, 2.6)	2.5 (2.2, 2.8)	0.024	0.387
				3α,5β,17,20α-PT/17-OH-20α-DHP, C								15 (11, 19)	9.4 (7.9, 11)	0.099	0.054
				3α,5β-THA)/A							10^3^	61 (49, 74)	78 (67, 90)	0.058	0.168
			**↑**	**3α/β,5β-THA, C/A**								**26 (19, 35)**	**47 (39, 57)**	**0.152**	**0.019**
			**↑**	**3α/β,5β,17β-ADC/A**								**10 (7.7, 14)**	**17 (14, 21)**	**0.134**	**0.036**
				11β-OH-3α,5β-THA/A							10^3^	43 (35, 50)	34 (28, 39)	0.05	0.183
				11β-OH-3α,5β-THA, C/A							10^3^	250 (170, 330)	220 (170, 280)	0.004	0.707
EXPLAINED VARIABLE				MS patient vs. control (LLR)											
				Not relevant											
EXPLAINING VARIABLES	**AKR1C1 vs. HSD17B2**		**↓**	**20α-DHPreg/Preg**	**−0.577**	−4.38 **	**−0.728**	**−0.389**	−5.06 **			**3.4 (2.9, 3.9)**	**2.7 (2.3, 3)**	**0.076**	**0.109**
			**↓**	**20α-DHPreg/Preg, C**								**7 (6.1, 8)**	**5.1 (4.7, 5.5)**	**0.22**	**0.005**
			**↑**	**20α-DHP/P**	**0.332**	2.46 *	**0.407**	**0.054**	**0.71**			**1.3 (0.88, 1.7)**	**2.2 (1.8, 2.8)**	**0.109**	**0.046**
			**↑**	**20α-DHPC/P**	**0.312**	3.3 **	**0.385**	**−0.083**	**−0.87**			**7.7 (4.1, 14)**	**13 (7.8, 20)**	**0.021**	**0.38**
				17-OH-20α-DHP/17-OH-P								0.55 (0.43, 0.68)	0.76 (0.65, 0.89)	0.079	0.092
			**↑**	**17-OH-20α-DHPC/17-OH-P**	**0.537**	7.77 **	**0.668**	**0.139**	**1.35**			**6.3 (4.3, 9.2)**	**11 (8.2, 15)**	**0.07**	**0.103**
				5α,20α-THP/5α-DHP								1.5 (1.3, 1.8)	1.7 (1.5, 2)	0.015	0.522
				5α,20α-THPC/5α-DHP								3.5 (2.4, 5)	3.9 (2.9, 5.3)	0.004	0.724
				3α,5α,20α-PD/3α,5α-THP								1.7 (1.2, 2.4)	1.6 (1.2, 2.2)	<0.001	0.905
				3α,5α,20α-PD/3α,5α-THP, C								4.7 (4.1, 5.4)	4.7 (4.3, 5.3)	<0.001	0.98
				3β,5α,20α-PD/3β,5α-THP								24 (18, 31)	22 (17, 27)	0.006	0.679
				3β,5α,20α-PD/3β,5α-THP, C								44 (37, 53)	45 (39, 51)	<0.001	0.963
				3α,5β,20α-PD/3α,5β-THP								1.8 (1.3, 2.4)	1.2 (0.95, 1.6)	0.059	0.188
				3α,5β,20α-PD/3α,5β-THP, C								1 (0.88, 1.3)	1.1 (0.93, 1.2)	<0.001	0.934
				3β,5β,20α-PD/3β,5β-THP, C								5.1 (4.2, 6.2)	5.3 (4.6, 6.3)	0.002	0.776
				3α,5α,17,20α-PT/3α,5α,17-PD, C								22 (16, 30)	21 (17, 27)	<0.001	0.864
			**↑**	**3α,5β,17,20α-PT/3α,5β,17-PD**	**0.433**	2.21 *	**0.586**	**0.312**	**1.76**			**32 (23, 44)**	**61 (45, 84)**	**0.135**	**0.042**
				3α,5β,17,20α-PT/3α,5β,17-PD, C								8.7 (7.1, 11)	10 (9, 12)	0.03	0.306
EXPLAINED VARIABLE				MS patient vs. control (LLR)	1.000	3.33 **	0.528								
				Explained variability = 31.2% (18% after cross-validation), Sensitivity = 0.786(0.634–0.938), Specificity = 0.727(0.464–0.99)											
EXPLAINING VARIABLES	**AKR1C2 vs. HSD17B2,6**	3α/β		3α,5α-THP/3β,5α-THP								1.7 (1.4, 2.1)	1.5 (1.3, 1.8)	0.018	0.461
			**↓**	**3α,5α-THP/3β,5α-THP, C**	**−0.220**	**−1.87**	**−0.343**	**−0.114**	−2.51 *			**0.82 (0.69, 1)**	**0.53 (0.47, 0.59)**	**0.22**	**0.003**
			**↓**	**3α,5β-THP/3β,5β-THP, C**	**−0.435**	−5.75 **	**−0.677**	**−0.169**	−2.8 *			**7.2 (6.4, 8)**	**4.9 (4.5, 5.3)**	**0.311**	**<0.001**
				3α,5α,20α-PD/3β,5α,20α-PD							10^3^	95 (65, 140)	110 (76, 150)	0.003	0.756
				3α,5α,20α-PD/3β,5α,20α-PD, C							10^3^	58 (43, 80)	58 (47, 72)	<0.001	0.992
			**↓**	**3α,5β,20α-PD/3β,5β,20α-PD, C**	**−0.506**	−6.05 **	**−0.788**	**−0.209**	−5.29 **			**1.7 (1.4, 1.9)**	**1 (0.89, 1.1)**	**0.3**	**<0.001**
			**↓**	**3α,5α,17,20α-PT/3β,5α,17,20α-PT**	**−0.482**	−4.84 **	**−0.750**	**−0.174**	−3.13 **			**1.2 (1, 1.5)**	**0.75 (0.63, 0.88)**	**0.176**	**0.011**
			**↓**	**3α,5α,17,20α-PT/3β,5α,17,20α-PT, C**								**13 (9.2, 19)**	**6.2 (4.7, 8.1)**	**0.147**	**0.021**
				3α,5α-THA/3β,5α-THA								1.8 (1.5, 2)	2 (1.8, 2.3)	0.023	0.36
				3α,5α-THA/3β,5α-THA, C								2.6 (2.4, 2.9)	2.7 (2.5, 2.9)	0.001	0.848
				3α,5β-THA/3β,5β-THA, C								3.4 (2.9, 4.1)	2.7 (2.4, 3.1)	0.067	0.117
				3α,5α,17β-AD/3β,5α,17β-AD								2.7 (2.2, 3.4)	3 (2.6, 3.6)	0.009	0.586
				3α,5α,17β-AD/3β,5α,17β-AD, C								0.52 (0.45, 0.59)	0.51 (0.46, 0.57)	<0.001	0.926
			**↓**	**3α,5β,17β-AD/3β,5β,17β-AD, C**	**−0.273**	−2.21 *	**−0.425**	**−0.136**	−2.26 *			**15 (13, 18)**	**12 (11, 13)**	**0.084**	**0.073**
				11β-OH-3α,5α-THA/11β-OH-3β,5α-THA								25 (20, 31)	22 (19, 26)	0.013	0.509
			**↓**	**11β-OH-3α,5α-THA/11β-OH-3β,5α-THA, C**	**−0.335**	−3.08 **	**−0.521**	**−0.129**	−2.16 *			**34 (29, 40)**	**24 (21, 28)**	**0.134**	**0.033**
		3α/oxo		3α,5α-THP/5α-DHP								1.7 (1.3, 2.1)	2.2 (1.8, 2.6)	0.048	0.237
				3α,5α-THPC/5α-DHP								77 (51, 120)	71 (51, 100)	0.002	0.833
			**↑**	**3α,5α,20α-PD/5α,20α-THP**	**0.315**	2.86 *	**0.490**	**0.116**	2.24 *			**1.8 (1.3, 2.4)**	**2.3 (1.8, 2.9)**	**0.024**	**0.364**
				3α,5α,20α-PD/5α,20α-THP, C								86 (67, 110)	96 (81, 110)	0.01	0.565
				3α,5β,20α-PD/5β,20α-THP, C								50 (40, 61)	35 (30, 41)	0.103	0.056
			**↑**	**3α,5α-THA/5α-DHA**								**1.7 (1.5, 1.9)**	**2.5 (2.3, 2.8)**	**0.297**	**0.001**
			**↑**	**3α,5α-THAC/5α-DHA**							**10^−3^**	**3.6 (2.8, 4.6)**	**5.9 (4.9, 7)**	**0.138**	**0.028**
EXPLAINED VARIABLE				MS patient vs. control (LLR)	1.000	4.88 **	0.631								
				Explained variability = 39.8% (31.8% after cross-validation), Sensitivity = 0.767(0.615–0.918), Specificity = 0.7(0.416–0.984)											
EXPLAINING VARIABLES	**AKR1C3 vs. HSD17B2**			Adiol/DHEA								0.29 (0.26, 0.33)	0.29 (0.26, 0.32)	<0.001	0.934
				Adiol/DHEA, C								0.44 (0.36, 0.55)	0.36 (0.3, 0.42)	0.034	0.27
				3β,7α,17β-AT/7α-OH-DHEA								0.32 (0.28, 0.38)	0.32 (0.29, 0.36)	<0.001	0.98
			**↑**	**3β,7β,17β-AT/7β-OH-DHEA**								**0.33 (0.28, 0.4)**	**0.48 (0.41, 0.55)**	**0.129**	**0.029**
			**↑**	**T/A**								**0.18 (0.16, 0.22)**	**0.25 (0.22, 0.29)**	**0.114**	**0.047**
			**↑**	**5α-DHT/5α-DHA**	**0.791**	4.06 **	**0.826**	**0.392**	3.73 **			**0.72 (0.61, 0.85)**	**1.1 (0.93, 1.2)**	**0.181**	**0.012**
			**↑**	**5α-DHTC/5α-DHA**	**0.613**	2.25 *	**0.634**	**0.282**	**1.83**			**6.2 (5.2, 7.5)**	**8.8 (7.5, 10)**	**0.112**	**0.046**
				3α,5α,17β-AD/3α,5α-THA							10^3^	140 (120, 170)	130 (120, 150)	0.01	0.573
				3α,5α,17β-AD/3α,5α-THA, C							10^3^	39 (33, 45)	32 (28, 36)	0.054	0.178
				3β,5α,17β-AD/3β,5α-THA							10^3^	82 (60, 110)	96 (78, 120)	0.012	0.525
				3β,5α,17β-AD/3β,5α-THA, C							10^3^	210 (180, 240)	200 (180, 220)	0.003	0.735
				3α,5β,17β-AD/3α,5β-THA, C							10^3^	110 (100, 130)	110 (97, 120)	0.008	0.603
				3β,5β,17β-AD/3β,5β-THA, C							10^3^	22 (19, 26)	25 (22, 28)	0.02	0.413
EXPLAINED VARIABLE				MS patient vs. control (LLR)	1.000	2 *	0.500								
				Explained variability = 25% (17% after cross-validation), Sensitivity = 0.679(0.506–0.852), Specificity = 0.455(0.16–0.749)											

aR−Component loadings expressed as a correlation coefficients with predictive component, * *p* < 0.05, ** *p* < 0.01, blogarithm of likelihood ratio (probability of belonging to the selected group/probability of not belonging to the selected group); Pregnenolone (Preg), Pregnenolone sulfate (PregS), 20α-Dihydropregnenolone (20α-DHPreg), 20α-Dihydropregnenolone sulfate (20α-DHPregS), 17-Hydroxypregnenolone (17-OH-Preg), 17-Hydroxypregnenolone sulfate (17-OH-PregS), 16α-Hydroxypregnenolone (16α-OH-Preg), Dehydroepiandrosterone (DHEA), DHEA sulfate (DHEAS), 7α-Hydroxy-DHEA (7α-OH-DHEA), 7β-Hydroxy-DHEA (7β-OH-DHEA), Androstenediol (Adiol), Androstenediol Sulfate (AdiolS), 5-Androstene-3β,7α,17β-triol (3β,7α,17β-AT), 5-Androstene-3β,7β,17β-triol (3β,7β,17β-AT), 5-Androstene-3β,16α,17β-triol (3β,16α,17β-AT), Conjugated 5-androstene-3β,16α,17β-triol (3β,16α,17β-ATC), Progesterone (P), 20α-Dihydroprogesterone (20α-DHP), Conjugated 20α-dihydroprogesterone (20α-DHPC), 17-Hydroxyprogesterone (17-OH-P), 17,20α-Dihydroxy-4-pregnene-3-one (17-OH-20α-DHP), Conjugated 17,20α-dihydroxy-4-pregnene-3-one (17-OH-20α-DHPC), 16α-Hydroxyprogesterone (16α-OH-P), Androstenedione (A), Testosterone (T), 5α-Dihydrotestosterone (5α-DHT), Conjugated 5α-dihydrotestosterone (5α-DHTC), Estradiol (E2), 5α-Dihydroprogesterone (5α-DHP), Allopregnanolone (3α,5α-THP), Allopregnanolone sulfate (3α,5α-THPC), Isopregnanolone (3β,5α-THP), Isopregnanolone sulfate (3β,5α-THPC), Pregnanolone (3α,5β-THP), Conjugated pregnanolone (3α,5β-THPC), Conjugated epipregnanolone (3α,5β-THPC), 17-Hydroxyallopregnanolone sulfate (3α,5α,17-PDC), 17-Hydroxypregnanolone (3α,5β,17-PD), Conjugated 17-hydroxypregnanolone (3α,5β,17-PDC), 5α,20α-Tetrahydroprogesterone (5α,20α-THP), Conjugated 5α,20α-tetrahydroprogesterone (5α,20α-THPC), 5α-Pregnane-3α,20α-diol (3α,5α,20α-PD), Conjugated 5α-pregnane-3α,20α-diol (3α,5α,20α-PDC), 5α-Pregnane-3β,20α-diol (3β,5α,20α-PD), Conjugated 5α-pregnane-3β,20α-diol (3β,5α,20α-PDC), Conjugated 5β,20α-tetrahydroprogesterone (5β,20α-THPC), 5β-Pregnane-3α,20α-diol (3α,5β,20α-PD), Conjugated 5β-pregnane-3α,20α-diol (3α,5β,20α-PDC), 5β-Pregnane-3β,20α-diol (3β,5β,20α-PD), Conjugated 5β-pregnane-3β,20α-diol (3β,5β,20α-PDC), 5α-Pregnane-3α,17,20α-triol (3α,5α,17,20α-PT), Conjugated 5α-pregnane-3α,17,20α-triol (3α,5α,17,20α-PTC), 5α-Pregnane-3β,17,20α-triol (3β,5α,17,20α-PT), Conjugated 5α-pregnane-3β,17,20α-triol (3β,5α,17,20α-PTC), 5β-Pregnane-3α,17,20α-triol (3α,5β,17,20α-PT), Conjugated 5β-pregnane-3α,17,20α-triol (3α,5β,17,20α-PTC), 5α-Androstane-3,17-dione (5α-DHA), Androsterone (3α,5α-THA), Androsterone sulfate (3α,5α-THAC), Epiandrosterone (3β,5α-THA), Epiandrosterone sulfate (3β,5α-THAC), Etiocholanolone (3α,5β-THA), Etiocholanolone sulfate (3α,5β-THAC), Epietiocholanolone sulfate (3β,5β-THAC), 5α-Androstane-3α,17β-diol (3α,5α,17β-AD), Conjugated 5α-androstane-3α,17β-diol (3α,5α,17β-ADC), 5α-Androstane-3β,17β-diol (3β,5α,17β-AD), Conjugated 5α-androstane-3β,17β-diol (3β,5α,17β-ADC), Conjugated 5β-androstane-3α,17β-diol (3α,5β,17β-ADC), Conjugated 5β-androstane-3β,17β-diol (3β,5β,17β-ADC), Cortisol (F), Cortisone (E), Corticosterone (B), 11β-Hydroxyandrostenedione (11β-OH-A), 11β-Hydroxyandrosterone (11β-OH-3α,5α-THA), 11β-Hydroxyandrosterone sulfate (11β-OH-3α,5α-THAC), 11β-Hydroxyepiandrosterone (11β-OH-3β,5α-THA), 11β-Hydroxyepiandrosterone sulfate (11β-OH-3β,5α-THAC), 11β-Hydroxyetiocholanolone (11β-OH-3α,5β-THA), 11β-Hydroxyetiocholanolone sulfate (11β-OH-3α,5β-THAC).

## Data Availability

The original contributions presented in the study are included in the article, further inquiries can be directed to the corresponding authors.

## Abbreviations

11β-OH-3α,5α-THA	11β-Hydroxyandrosterone
11β-hydroxyandrostenedione	11β-OH-A
16α-OH-P	16α-Hydroxyprogesterone
16α-OH-Preg	16α-Hydroxypregnenolone
17-OH-P	17-Hydroxyprogesterone
17-OH-Preg	17-Hydroxypregnenolone
17-OH-20α-DHP	17α-Hydroxy-20α-dihydroprogesterone
5α-DHA	5α-Androstane-3,17-dione
5α-DHT	5α-Dihydrotestosterone
3α,5α,17-PD	17-Hydroxyallopregnanolone
3α,5α,17,20α-PT	5α-Pregnane-3α,17,20α-triol
3α,5α,20α-PD	5α-Pregnane-3α, 20α-diol
3α,5α-THA	Androsterone
3α,5α-THP	3α,5α-Tetrahydroprogesterone, allopregnanolone
3β,5α,17,20α-PT	5α-Pregnane-3β,17,20α-triol
3β,5α-THA	Epiandrosterone
3α,5β,17,20α-PT	5β-Pregnane-3α,17,20α-triol
3α,5β,17-PD	17-Hydroxypregnanolone
3α,5β-THA	Etiocholanolone
3α,5β-THA	Epietiocholanolone
8-iso-PGF2α	8-iso-Prostaglandin F_2α_
σ_1_Rs	Sigma-1 receptors
A	Androstenedione
ACTH	Adrenocorticotropic hormone
AD	Androstanediol
ADIOL	Androstenediol
AKR1C1	Aldo-keto reductase family 1 member C1
AKR1C2	Aldo-keto reductase family 1 member C2
AKR1C3	Aldo-keto reductase family 1 member C3
AKR1D1	5β-Reductase
AMPARs	α-Amino-3-hydroxy-5-methyl-4-isoxazolepropionic acid receptors
ANOVA	Analysis of variance
AT	Androstenetriol (5-androstene)
BBB	Blood-brain barrier
CI	Confidence interval
CNS	Central nervous system
CRH	Corticotropin-releasing hormone
CYP11A1	Cholesterol desmolase
CYP11B1	11β-Hydroxylase
CYP17A1	C17-hydroxylase-C17,20-lyase
CYP19A1	Aromatase
CYP21A2	21-Hydroxylase
CYP3A4	Cytochrome P450 3A4
CYP3A7	Cytochrome P450 3A7
CYP7B1	7α-hydroxylase
DHEA	Dehydroepiandrosterone
DHEAS	Dehydroepiandrosterone sulfate
DHP	Dihydroprogesterone
EDSS	Expanded Disability Status Scale
ERα	Estrogen receptors α
ERβ	Estrogen receptors β
FSH	Follicle-stimulating hormone
GABA	γ-Aminobutyric acid
GABA_A_Rs	Type A γ-aminobutyric acid receptors
GR	Glucocorticoid receptors
GC-MS/MS	Gas chromatography tandem mass spectrometry
GPER	G protein-coupled estrogen receptors
HPAA	Hypothalamic-pituitary-adrenal axis
HSD3B1	Type 1 3β-Hydroxysteroid dehydrogenase
HSD3B2	Type 2 3β-Hydroxysteroid dehydrogenase
HSD3Bs	Type 1 and 2 3β-Hydroxysteroid dehydrogenases
HSD11B1	Type 1 11β-Hydroxysteroid dehydrogenase
HSD11B2	Type 2 11β-Hydroxysteroid dehydrogenase
HSD17B2	Type 2 17β-Hydroxysteroid dehydrogenase
HSD17B3	Type 3 17β-Hydroxysteroid dehydrogenase
HSD17B6	Type 6 17β-Hydroxysteroid dehydrogenase (3α/β epimerase)
HPT9_R	9-Hole Peg Test for right hand
HPT9_L	9-Hole Peg Test for left hand
IFN-γ	Interferon γ
IL-1	Interleukin 1
IL-2	Interleukin 2
IL-4	Interleukin 4
IL-6	Interleukin 6
IL-10	Interleukin 10
IL-12	Interleukin 12
IL-17	Interleukin 17
LLR	Logarithm of likelihood ratio
mRNA	Messenger ribonucleic acid
NASs	Neuroactive steroids
NMDA	N-methyl-D-aspartate
NMDARs	N-methyl-D-aspartate receptors
OPLS	Orthogonal predictions to latent structure
P	Progesterone
PD	Pregnanediol
PGF_2α_	Prostaglandin F_2α_
PNS	Peripheral nervous system
Preg	Pregnenolone
PregS	Pregnenolone sulfate
PT	Pregnanetriol
ROS	Reactive oxygen species
SRD5A1	Type 1 5α-reductase
SRD5A2	Type 2 5α-reductase
SULT2A1	Steroid sulfotransferase type 2A1
STS	Steroid sulfatase
T	Testosterone
T25FWT	Timed 25-foot walk
THP	Tetrahydroprogesterone
TNF-α	Tumor necrosis factor α
TRPC5s	Short transient receptor potential channels 5
TRPV1s	Vanilloid receptors
TRPM3s	Melastatin receptors
The letter C after the abbreviation of a steroid indicates its conjugated form	
The “, C” after the molar ratio of a steroid indicates that they are its conjugated forms.	
